# Measuring vision using innate behaviours in mice with intact and impaired retina function

**DOI:** 10.1038/s41598-019-46836-y

**Published:** 2019-07-17

**Authors:** R. Storchi, J. Rodgers, M. Gracey, F. P. Martial, J. Wynne, S. Ryan, C. J. Twining, T. F. Cootes, R. Killick, R. J. Lucas

**Affiliations:** 1 Faculty of Biology, Medicine and Health, University of Manchester, Manchester, UK; 2 Department of Mathematics and Statistics, Lancaster University, Lancaster, UK; 3 School of Computer Science, University of Manchester, Manchester, UK

**Keywords:** Retina, Sensorimotor processing

## Abstract

Measuring vision in rodents is a critical step for understanding vision, improving models of human disease, and developing therapies. Established behavioural tests for perceptual vision, such as the visual water task, rely on learning. The learning process, while effective for sighted animals, can be laborious and stressful in animals with impaired vision, requiring long periods of training. Current tests that that do not require training are based on sub-conscious, reflex responses (e.g. optokinetic nystagmus) that don’t require involvement of visual cortex and higher order thalamic nuclei. A potential alternative for measuring vision relies on using visually guided innate defensive responses, such as escape or freeze, that involve cortical and thalamic circuits. In this study we address this possibility in mice with intact and degenerate retinas. We first develop automatic methods to detect behavioural responses based on high dimensional tracking and changepoint detection of behavioural time series. Using those methods, we show that visually guided innate responses can be elicited using parametisable stimuli, and applied to describing the limits of visual acuity in healthy animals and discriminating degrees of visual dysfunction in mouse models of retinal degeneration.

## Introduction

Rodents, and in particular mice, are increasingly applied to understanding the physiology and neural computations underlying vision. Rodent models of ocular diseases are also an important tool to develop therapies at preclinical level. In this field a great body of work has been dedicated to developing rodent models that capture the critical aspects of human diseases and there are currently >100 different types of visually impaired mouse^1^. A particular focus has been devoted to using these models to develop treatments based on optogenetics, stem cell therapies and gene therapies for such incurable conditions as retinitis pigmentosa and Leber congenital amaurosis^2–4^.

A crucial step in describing degeneration and establishing therapeutic efficacy in such pre-clinical studies is assessment of visual function, commonly by electrophysiological and/or behavioural approaches. Electrical recordings of neural activity in the retina and in the brain can be very useful in revealing physiological responses to visual stimuli^5,6^. However, they don’t directly measure integrated visual performance or perceptual components of vision^7^. Many commonly used behavioural assays of visual function, such as pupil constriction^8^, optokinetic and optomotor reflexes^9,10^, suffer from the same weakness; being sub-conscious, reflex responses whose activity is only indirectly related to perceptual vision. All current tests that allow measurement of perceptual, cortically modulated vision rely on learning^11–13^. The most commonly used, the visual water task, has been effectively and widely employed to measure spatial acuity and brightness discrimination in mice with intact visual systems. However in animal models of moderate or severe retinal degeneration this task can only be painstakingly learnt through several weeks of intensive training^7^. This process is time consuming and can be significantly stressful for the animals^14^. Furthermore the visual water task can introduce additional sources of variability including the effects of repeated handling^15^ and the learning process itself^16,17^.

The aim of this work is to develop a rapid assay of visual stimulus detection, suitable for mice with intact and degenerate retinas, which doesn’t rely on training. To meet these requirements we investigate the possibility of using innate behaviours instead of learned tasks. Visually evoked changes in exploratory behaviour have been applied to this problem^6,18^, but such tests are relatively low throughput and we were interested in the possibility of exploiting recent advances in understanding of rapid defensive behaviours as an alternative. Behaviourally salient visual stimuli, such as a looming dark spot^19,20^ or a full field flash of light^21^, can reliably trigger different behavioural responses. Several studies aimed at elucidating the circuits involved in controlling these behaviours revealed a substantial involvement of primary visual cortex^21,22^ and first and higher order thalamic nuclei^23,24^ as well as spatial memory^25^. This indicates that these behaviours are not simply sub-conscious reflexes and instead rely on processing of higher order image forming pathways and integration with the limbic system. These behaviours are rapidly induced and short lived, meaning that they can, in theory, be evoked multiple times in a single experimental session. However so far such innate responses haven’t been used systematically to assay visual ability, nor have they been tested in animals with mild or severe visual impairments. Therefore it is not clear to what extent these responses could be used to discriminate different degrees of retinal function.

We show that measuring innate responses to experimentally controlled changes in the visual scene (such as light intensity or spatial frequency) represents an efficient strategy for measuring the limits of visual acuity in mice with an intact visual system. Importantly innate responses are partially maintained in animals with impaired vision and can be used to discriminate different levels of vision loss.

## Results

### Behavioural responses in visually intact mice

**Figure 1:**
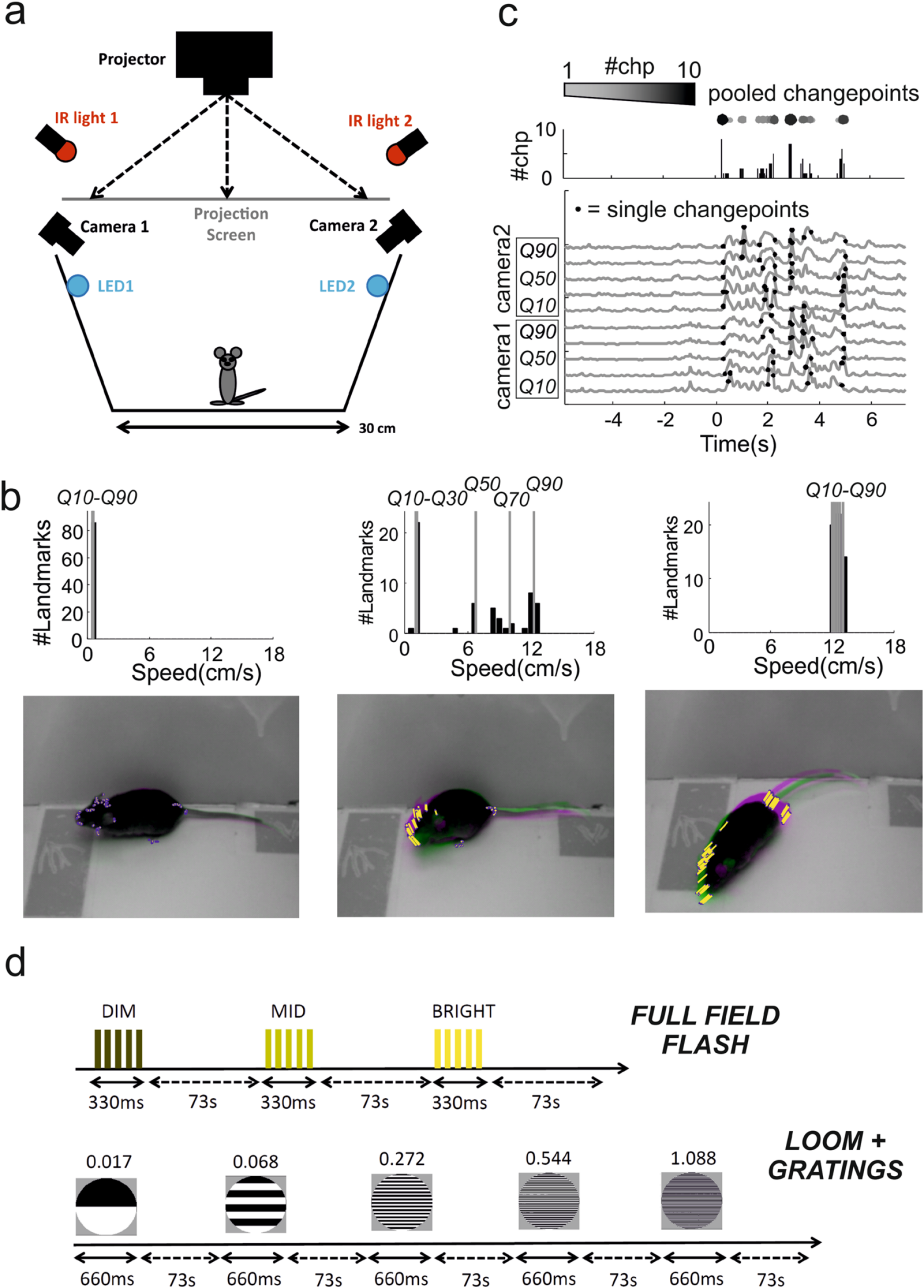
Behavioural arena, data analyses and experimental protocols. (**a**) Schematic of the open field arena used for all tests (dimension: 30 × 30 cm). Power LEDs were used to provide diffuse illumination for the flashes while a rear projection screen was used to deliver the looming stimuli. (**b**) Images of a mouse in the arena (bottom) over successive frames (superimposed pink and green images) during periods of quiescence (left), head movement (middle) and locomotion (right). Landmarks were automatically identified on the mouse’s body (blue dots on images at bottom) and the speed of each landmark calculated as proportional to the change in position from previous frame (visualised as yellow lines). Distribution of speed across landmarks was then calculated for each frame (top panels) and 10^th^, 30^th^, 50^th^, 70^th^, 90^th^ speed quantiles (respectively Q10 to Q90) used for subsequent analyses. (**c**) Behavioural time series showing velocity of landmarks (Q10–90) extracted from two cameras (camera 1 and 2, bottom panel; visual stimulus at time 0). Changepoint detection was run independently on each series (single changepoints depicted by black dots) and identified changepoints were then summed for each frame across those series (top panels, “pooled changepoints”) and used for changepoint statistics throughout the study. (**d**) Schematic of the two stimulation protocols used for full field flash and pattern detection. In both protocols the order of each stimulus is block randomised (note: each block is represented by the list of all the distinct stimuli for that protocol).

We first set out to apply innate responses to measure visual capabilities in mice with an intact visual system. To this end, we developed an algorithm for tracking many landmarks on an animal’s body in order to improve our ability to detect and characterise behavioural responses in an open field arena (Fig. 1a,b, see Methods for details). For each pair of consecutive frames the change in location of landmarks was used to calculate speed of motion (Fig. 1b, lower panels), and the distribution of velocities across landmarks divided into quantiles (Fig. 1b, upper panels) and used to generate a multidimensional time series representing the movements of different body parts (Fig. 1c). By plotting distinct behavioural time series for each quantile we could then detect movie frames in which movement was restricted to parts of the body (e.g. head movements or rearing; Fig. 1b, middle panel), as well as full body movements (e.g. locomotion; Fig. 1b, right panel).

We considered two ways of using the tracking data to measure behavioural responses to a visual stimulus. In the simplest, we could use the estimates of landmark speed to identify increases or decreases in movement. In the second, we established changepoint analysis as a method to identify responses, and to quantify the frequency and reliability of visually induced behaviours. Changepoint analysis is used to detect if and when the properties of a time series change. By applying this technique to the behavioural time series, we can identify when a mouse’s behaviour changes. We reasoned that if visual stimuli affect behaviour then we would expect higher rates of behavioural changes to occur after the onset of those stimuli (shown in Fig. 1c). Thus we used Pruned Exact Linear Time algorithm (PELT)^26^ to detect changes in speed in our behavioural time series. This method is computationally efficient and can analyse dozens of our time series in less than a second. The PELT algorithm uses a penalty term to prevent overfitting changepoints (see Methods). This penalty term determines how much a changepoint must improve the model fit in order to be included. Small values result in a high rate of changepoints and are suitable to detect small changes in movements while larger values will only detect more substantial changes. In order to identify a suitable value for our data we investigated how the penalty value affects changepoint detection using the CROPS algorithm (Haynes *et al*., 2017) and knee estimation^27^ (see Methods for details and Supplementary Fig. 1b,c).

We designed two sets of stimuli aimed at probing different levels of visual capabilities. For the first we presented the animals with sudden changes in ambient light (bursts of 5 × 30 ms flashes over 330 ms, Fig. 1d, “full field flash”) at three different magnitudes (see Table 1 for calibrated background and flash intensities). Since the flash stimulus produces a global change in ambient light no spatial discrimination is required. For the second set we generated a modified looming stimulus where the dark enlarging spot was replaced by a static grating whose average intensity was isoluminant with the grey background (Table 2; Spatial Frequency = 0.017, 0.068, 0.272, 0.544, 1.088 cycles/degree; stimulus duration: 660 ms; looming speed = 66 deg/s; Inter-Stimulus-Interval = 73 seconds; Fig. 1d, “loom + gratings”). By changing the spatial frequency of the grating we could then use this stimulus to probe spatial acuity. As background illumination in both experiments we used low photopic light (see Tables 1, 2).

**Table 1:** Background irradiance (log_10_ photon/cm^2^/s).

	S-cone opsin	Melanopsin	Rhodopsin	M-cone opsin
Full Field Flash Contrast Response	10.0552	12.765	12.8345	13.0188
Gratings Looming for Spatial Acuity	10.6106	13.2171	13.2868	13.4712
Full Field Flash Contrast Response (RD1 mice)	9.16516	12.0543	12.1219	12.2971

**Table 2:** Michelson Contrast (%).

Full Field Contrast Response	S-cone opsin	Melanopsin	Rhodopsin	M-cone opsin
DIM	32.8793	44.1403	35.2123	11.2034
MID	86.1193	89.1086	84.8028	56.2394
BRIGHT	99.4891	99.5334	99.3131	97.0982
**Gratings Looming for Spatial Acuity** ^*^	**S-cone opsin**	**Melanopsin**	**Rhodopsin**	**M-cone opsin**
White vs Grey	35.254	35.254	35.254	35.254
Black vs Grey	−87.113	−87.113	−87.113	−87.113
White vs Black	93.617	93.617	93.617	93.617
**Full Field Flash Contrast Response (RD1 mice)**	**S-cone opsin**	**Melanopsin**	**Rhodopsin**	**M-cone opsin**
DIM	79.1772	80.2339	73.7149	39.9271
MID	97.9658	97.6759	96.6435	87.1301
BRIGHT	99.9339	99.9088	99.8661	99.4359

^*^Michelson Contrast measured between white area of the gratings and grey background (White vs Grey), black area of the gratings and the grey background (Black vs Grey), white and black area of the gratings (White vs Black).

The mice expressed a variety of responses that differed both quantitatively and qualitatively. The full field stimulus evoked partial body movements, typically head movements or rearing, or full body changes in locomotor activity (respectively Movies 1, 2). The looming stimulus instead reduced the animal activity both when engaged in full body movements such as during locomotion and when the animal was performing more stationary exploration (respectively Movies 3, 4). Therefore the responses to flashes and looming were qualitatively different and consisted respectively of an increase and a decrease in movement speed.

**Figure 2:**
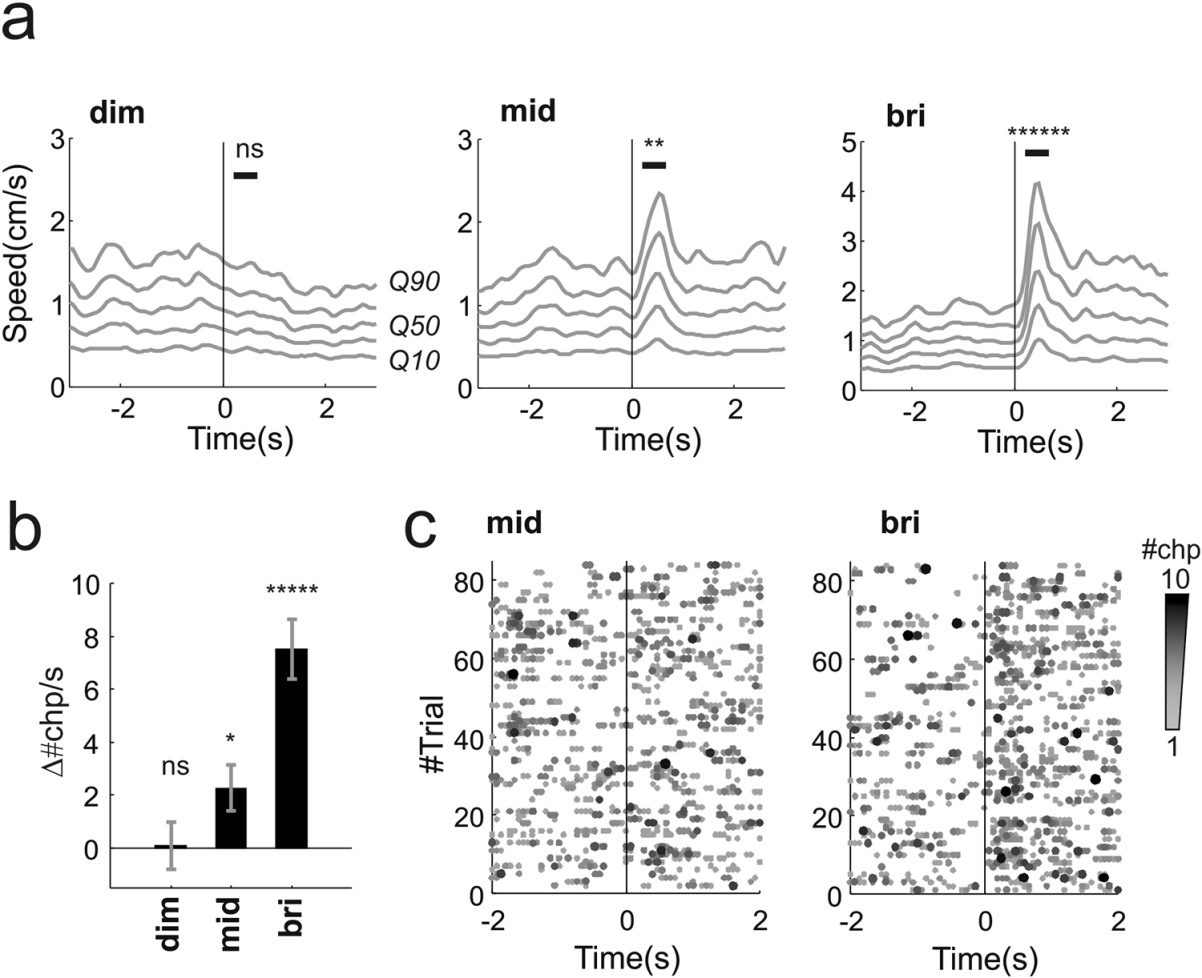
Behavioural responses to full field flashes in visually intact animals. (**a**) Movement responses to dim, mid and bright flashes (respectively left, middle and right panels; visual stimulus at time 0). Each grey line represents a different quantile of the speed distribution (10^th^, 30^th^, 50^th^, 70^th^, 90^th^ quantiles, respectively Q10 to Q90, averaged across the two cameras used to track behaviour, see also Fig. 1a–c and Methods). (**b**) Difference between changepoint rate after and before stimulus onset (calculated as *Δ#chp/s* = *#chp/s (pre) - #chp/s (post)*, see Methods; the rates were calculated in time windows of 0.6 s). (**c**) Behavioural changepoints for individual trials for mid and bright flashes (respectively left and right panels). For both flashes we collected 84 trials (each trial here reported as row along the y-axis) from 12 animals (7 trials/animal). The flash was delivered at time 0). For a given trial the number of changepoints occurring at the same time frame are colour coded gray-to-black as exemplified in Fig. 1c (“pooled changepoints”). *p < 0.05, **p < 0.0.01, ***p < 0.005, ****p < 0.001, *****p < 0.0005, ******p < 0.0001, ns = not significant.

The response to full field stimuli was apparent in analyses based upon speed of movement and changepoint detection. Thus, velocity of movement across landmarks increased following presentation of mid and bright, but not dim, flashes (Fig. 2a; p = 1, 0.006, 4 * 10^−7^ respectively dim, mid, bright flash; n = 84 trials from 12 animals, signtest). The average responses to highest intensity were substantially larger than to mid intensity levels (p = 0.013, z = 2.48, n = 84 “mid” & “bri” trials, ranksum test). We also found an increase in the number of changepoints after the stimulus onset compared to baseline activity, and this effect was flash intensity dependent (Fig. 2b, p < 0.0001, df = 2, χ^2^ = 27.57, n = 84, kruskalwallis test). The increase in number of changepoints was consistent across individuals for the bright flash (Supplementary Fig. 5a,b) while the mid flash revealed higher variability across subjects (Supplementary Fig. 5c). We wondered whether the larger average effects associated with high intensity was also due to an increase in reliability leading to more effective summation across trials. In other words whether part of the higher amplitude response to bright flash could be explained by the fact that dimmer flashes evoke both positive and negative responses that partially cancel out. In order to measure reliability of these responses we compared the rate of changepoints before and after stimulus onset on a single trial basis (calculated over time windows of 0.6 s duration). The rate increased in 74% trials and decreased in 11% trials for the highest intensity while for the mid-level intensity we found increments and decrements respectively in 45% and 27% trials. Therefore since the brightest flash produced a more consistent bias towards positive increments in changepoints we conclude that this stimulus also increased response reliability. The intensity dependent increase in reliability is also clearly apparent by comparing left and right panels in Fig. 2c, where we reported the changepoints detected for all individual trials for mid (left panel) and bright (right panel) flashes.

**Figure 3:**
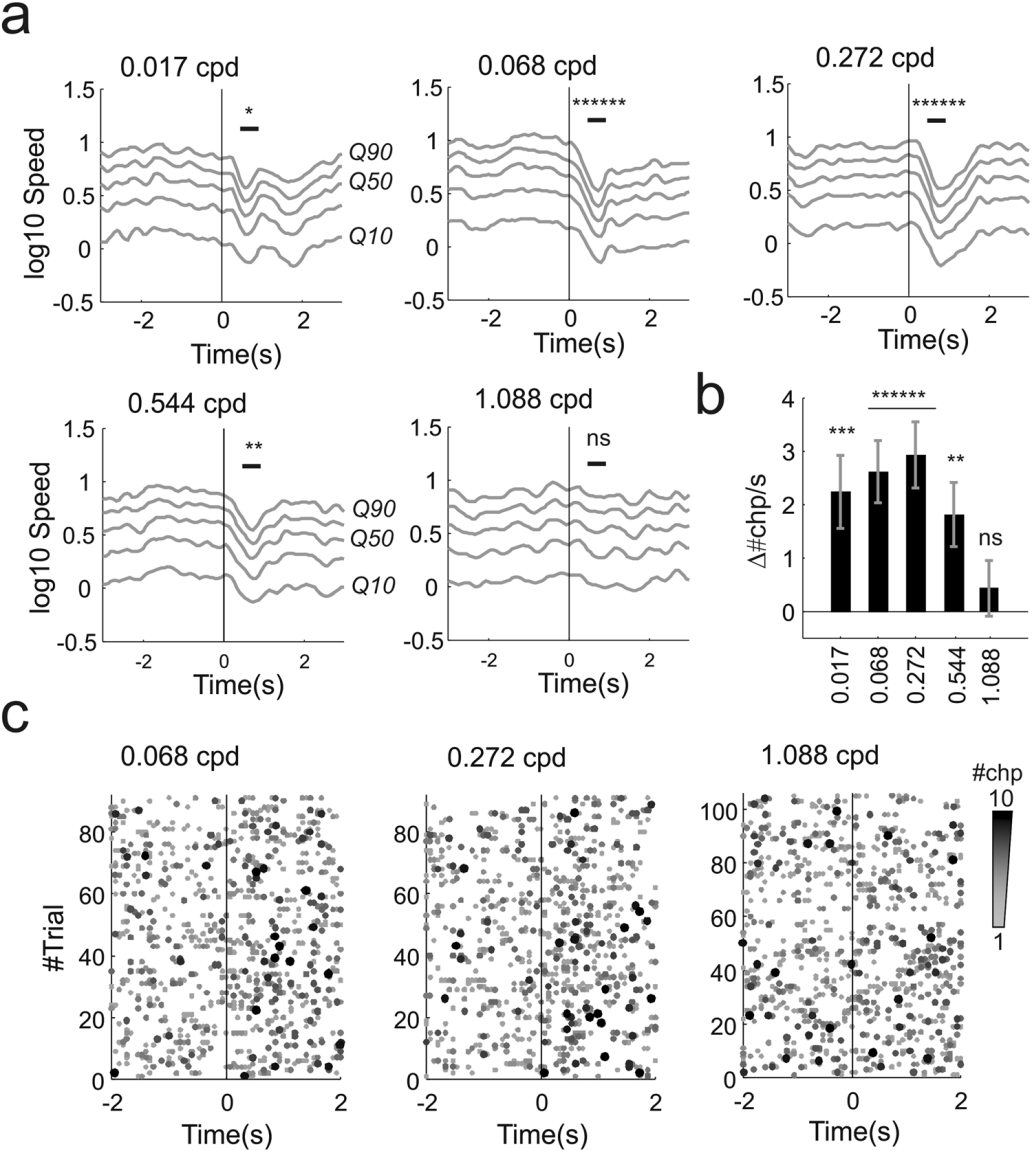
Behavioural responses to looming + gratings in visually intact animals. **(a**) Movement responses (in log scale for 10^th^, 30^th^, 50^th^, 70^th^, 90^th^ quantiles - respectively Q10 to Q90 - averaged across two cameras) to all spatial frequencies tested for the spatial acuity stimulus represented in Fig. 1d (the spatial frequency for each panel is indicated in cycles/degree in the top left corner of each panel; visual stimulus at time 0). Reduction in movement was significant for all frequencies apart from the highest (1.088 cycles/degree). (**b**) Differences in changepoint rate before and after stimulus onset across all spatial frequencies tested (mean ± sem; spatial frequencies are indicated as cycles/dregrees on the x-axis). (**c**) Behavioural changepoints for individual trials at different spatial frequencies (0.068, 0.272, 1.088 cycles/degree; 91, 91, 105 trials, 7 trials/animal; visual stimulus delivered at time 0). For a given trial the number of changepoints occurring at the same time frame are colour coded gray-to-black as exemplified in Fig. 1c (“pooled changepoints”). *p < 0.05, **p < 0.01, ***p < 0.005, ****p < 0.001, *****p < 0.0005, ******p < 0.0001, ns = not significant.

The looming stimuli elicited freezing-like reductions in activity across a wide range of frequencies. This effect could be observed from an average reduction in speed that was significant up to 0.544 cpd but not at 1.088 cycles/degrees (p = 0.021, 5 * 10^−5^, 5 * 10^−5^, 0.0063, 0.558 respectively 0.017, 0.068, 0.272, 0.544, 1.088 cycles/degrees; n = 91, 91, 91, 105, 105 trials from 15 animals, signtest; Fig. 3a). The changepoint analysis replicated the results obtained by measuring movement speed as significant responses could be measured up to 0.544 cycles/degree (Fig. 3b). The most reliable response occurred with the intermediate spatial frequency gratings 0.068 and 0.272 cycles/degree in which changepoint rate increased respectively in 61% and 65% trials and decreased in 30% and 26%. This effect can be observed in Fig. 3c where a clear increase in changepoint rate after stimulus onset (at time 0) is apparent at 0.068 and 0.272 cycles/degree (left and middle panel) but not at 1.088 cycles/degree (right panel). The highest reproducibility at 0.272 cycles/degree is consistent with peak in contrast sensitivity recorded with visual water task^11^. For spatial frequencies in this high sensitivity range responses were also reliable across individuals (Supplementary Fig. 5d,e) while, for spatial frequencies around the spatial acuity threshold (0.544–1.088 cycles/degree), responses were more variable across subjects (Supplementary Fig. 5f). Importantly, the threshold acuity found here (0.544 cpd) is higher than that defined in cortically lesioned animals (<0.3 cycles/degree with visual water task^28^) suggesting that visual cortex is involved in driving these behavioural responses. This possibility is also consistent with the described positive cortical modulation of looming evoked responses in superior colliculus^22^. In this study the authors demonstrated that primary visual cortex exerts a positive gain modulation of looming evoked firing rate in superior colliculus. Therefore it is possible that a similar modulation could enhance collicular responses to the highest spatial frequency and increase the probability of a behavioural response. However a conclusive proof of cortical involvement in driving innate freeze-like responses to high spatial frequency would require experimental manipulation of cortical activity. These experiments could be done either by lesioning the visual cortex or by transiently blocking cortical activity (either pharmacologically, or by using optogenetics or chemogenetics in transgenic animals).

Many of the previous studies on looming evoked defensive responses in mice employed a black looming disc^19,20,24,25,29–31^. Therefore we wondered whether the flashes or the loom + gratings evoked responses that were comparable to those expressed by the “standard” black looming stimulus. Since several of those studies focussed on escape responses^19,25,29^ we performed an additional set of experiments by adding a shelter to provide an escape route for the animal.

We found that the bright flash never evoked a clear run to the shelter (n = 0/22 trials, data from 11 animals; we recorded 2 trials per animal) and instead animals expressed diverse increases in movement speed that included enhanced locomotion or rearing (see Movie 9). Both looming + gratings and “standard” black looming typically evoked transient freeze followed by escape to the shelter (13/22 and 12/22 trials respectively loom + gratings & black looming; see Movie 10) and less commonly immediate escape (1/22 and 3/22 trials respectively loom + gratings & black looming; see Movie 11) or freeze (3/22 and 4/22 trials respectively loom + gratings & black looming). The qualitative difference between flash and looming is also apparent from individual trials (see Supplementary Fig. 2a) where, compared with flashes, black looming and loom + gratings alike elicit both higher and lower peaks speeds (Supplementary Fig. 2b) associated respectively with escape and freeze. In order to objectively test the possibility that behaviours expressed by flash and looming are largely separable we also performed a principal component analysis on the speed time series. We found that the first component could readily separate flash and looming stimuli on individual trials basis (Supplementary Fig. 2c; p = 5 * 10^−6^, 1 * 10^−6^, respectively for flash vs loom + gratings and flash vs black looming; two sample Kolmogorov-Smirnov goodness-of-fit test) while the score distribution for the first component were largely overlapping for black looming and loom + gratings (p = 0.33 for loom + gratings vs black looming; two sample Kolmogorov-Smirnov goodness-of-fit test). A clear difference between flash and looming evoked behaviours could also be observed from the average speed responses (Supplementary Fig. 2d).

These results indicate that we can effectively use parametrised stimuli (i.e. stimuli in which we can systematically vary relevant visual parameters, such as flash intensity or gratings spatial frequency) to measure different visual abilities, ranging from coarse light detection to noise-limited spatial acuity, in mice with intact retinas. Interestingly reactions to full field flashes are of opposite sign compared with looming gratings and produced qualitatively different behaviours, providing a novel case in which vision guiding of spontaneous behaviour is diverse yet systematic.

### Behavioural responses in mouse models of retinal degeneration

The use of innate responses as a tool to measure different levels of visual function in mice with intact visual system suggest that these responses could also be used to probe residual visual function in mouse models of retinal degeneration.

To investigate this possibility we first repeated our behavioural tests in rd1 mice where a mutation of the Pde6b gene substantially disrupts the rod phototransduction cascade^32^. These animals represent a model of severe retinal degeneration characterised by fast onset, in which the rod phototransduction cascade is non-functional from birth and cones undergo rapid progressive degeneration^33^. Adult rd1 mice (3–6 months) undergoing intensive training with visual water task (~3 weeks) can still learn a coarse dark/bright discrimination^7^, which is at least partly reliant on inner retina photoreception^34^, while detection of spatially structured patterns is abolished^7^. Previous work has shown that rd1 animals retain some innate light aversion however this behaviour is expressed only after prolonged light exposure (~10 minutes;^35^). Moreover, open field spontaneous exploratory behaviour is unaffected by exposure to bright light^5,6^. It is currently unknown whether their residual visual functionality can drive more transient behavioural responses resembling those we observed in visually intact animals; and, if that were the case, whether it is possible, based on these responses, to track the progress of visual degeneration.

To answer these questions we selected two cohorts of adult rd1 mice associated with different age groups hereinafter defined as “young” and “old” rd1 (n = 8,12 animals; 10.2 ± 0sd and 24 ± 1.75sd weeks). It was previously shown that cone density undergoes substantial changes between these ages^36^. The gradual loss of photoreceptor inputs then results in plastic changes and aberrant rhythmic activity at the level of the retinal ganglion cells^37^ targeting both visual thalamus and superior colliculus. Therefore behavioural responses, if at all present, would be expected to be more detectable in young animals.

**Figure 4:**
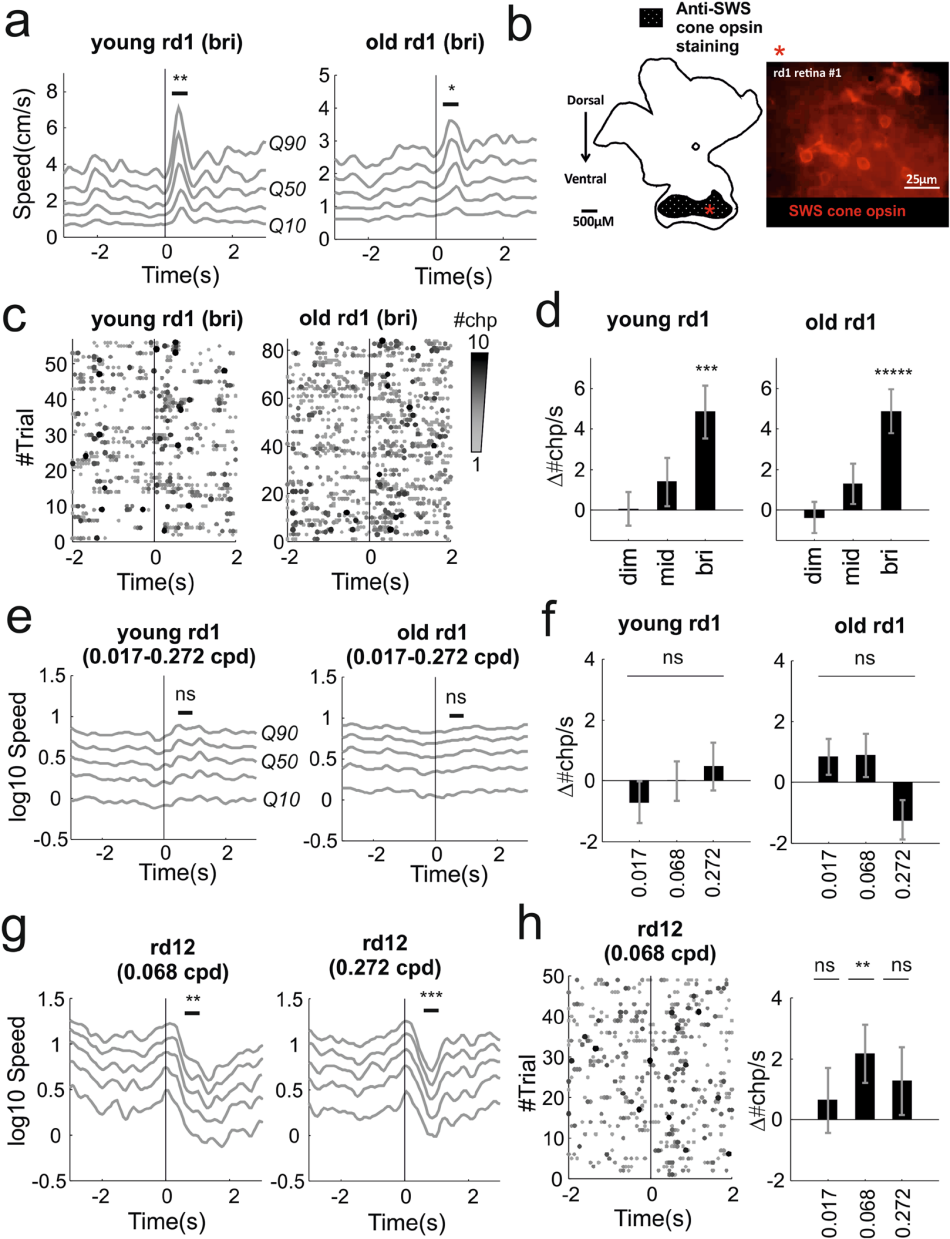
Behavioural responses to full field flashes and looming + gratings in mouse models of retinal degeneration. **(a**) Increase in speed after bright flash for young and old rd1 animals (respectively left and right panels). (**b**) Immunostaining of retinal wholemounts from “old” cohort of *rd1* mice revealed presence of surviving S-cones in peripheral ventral regions. A diagram showing pattern of anti-SWS cone opsin staining in a representative *rd1*retina (left), with an example micrograph of the anti-SWS cone staining we observed in the ventral retina shown on the right. Asterisk indicates location of micrograph in diagram of the retina. An expanded version of this figure is shown in Supplementary Fig. 7. (**c**) Behavioural changepoint for individual trials under bright flash stimuli for “young” and “old” animals (respectively left and right panels; 56, 84 trials collected from 8, 12 animals; each animals recorded for 7 trials; visual stimulus at time 0). (**d**) Increase in changepoint rate (mean ± sem) as function of flash intensity. (**e**) Looming did not evoke changes in speed in both groups. Speed, represented in log scale, was averaged across the spatial frequencies tested (0.017, 0.068 and 0.272 cycles/degree). (**f**) Changepoint rates did not significantly change after stimulus onset at all spatial frequencies tested (0.017, 0.068, 0.272 cycles/deg; mean ± sem). (**g**) rd12 expressed significant reductions in movement at 0.068 cycles/deg (left panel) and at 0.272 cycles/deg (right panel). (**h**) Behavioural changepoints for individual trials during looming gratings at 0.068 cycles/deg reveals repeatable responses (left). Changepoint rates as function of spatial frequencies (right panel; mean ± sem). *p < 0.05, **p < 0.01, ***p < 0.005, ****p < 0.001, *****p < 0.0005, ******p < 0.0001, ns = not significant.

As a first test we used the same full field flashes delivered to control mice but we reduced the background light intensity in order to maximise the contrast and effectiveness of the flash stimulus (see Tables 1, 2 for calibrated intensities). The highest intensity flash evoked a transient increase in activity in both age groups (Movies 5, 6, Fig. 4a; p = 0.011 and 0.0375, n = 56 and 84 trials, signtest). One potential explanation is that these responses could be driven by residual cones. This possibility is consistent with a previous study that described surviving cone photoreceptors up to 3 months of age^36^ and with immunohistochemistry analysis of retinas from animals in the “old” group that also provided positive evidence of residual S-cones (Fig. 4b**;** see also Supplementary Fig. 7). Alternatively these responses could be driven by intrinsically photosensitive retinal ganglion cells (ipRGCs), as melanopsin signalling is largely preserved in rd1 mice^38,39^. A further possibility is that both surviving cones and melanopsin contribute to these responses as ipRGCs mediated gain modulation of cone signalling has recently been described^40^. Lower intensities did not evoke significant responses (“young”: p = 0.894 and 0.504, n = 56 trials; “old”: p = 0.230 and 0.063, n = 84 trials; signtest for dim and mid intensity).

The response to the highest intensity flash was significantly larger in the young cohort indicating that this simple test allows to detect differences in coarse light detection among the two groups (p = 0.022, z = 2.3, n = 56 and 84, respectively “young” and “old” trials, ranksum test). Compared with control animals trial-to-trial reproducibility was reduced in both groups as changepoint rate increased in 57% & 52% and decreased in 16% & 23% of the trials (respectively young & old rd1; Fig. 4c). Individual variability in responses to the bright flash appeared larger that in visually intact animals (compare Supplementary Fig. 6a with Supplementary Fig. 5b). Like in control animals increase in changepoint rate was dependent on flash intensity (Fig. 4d, p = 0.021 and 0.004, df = 2, χ^2^ = 7.70 and 11.047, n = 56 and 84 trials, Kruskal-Wallis test respectively “young” and “old” groups).

We next used the looming stimuli in order to assess visual acuity. Since previous studies reported complete lack of spatial discrimination in these animals we focused on a reduced spatial frequency range (0.017, 0.068, 0.272 cycles/degree). Both age groups failed to exhibit significant responses to any of the stimuli presented (Fig. 4e,f**;** “young”: p = 0.689, 0.894, 0.141, n = 56 trials; “old”: p = 1, 0.445, 0.586, n = 84 trials; signtest at 0.017, 0.068, 0.272 cycles/degree). These negative results are consistent with data from both visual water task and optomotor assays showing loss of spatial discrimination in this genotype^7,10^.

We wondered whether behavioural responses to spatially structured stimuli such as our looming gratings could be observed in animals with less complete retinal degeneration. To investigate this possibility we used rd12 animals, which carry a mutation in the Rpe65 gene that disrupts recycling of the chromophore^41^. Previous results based on optokinetic reflex revealed detectable responses below 0.2 cycles/degrees in adult animals aged between 9 and 18 weeks^42^.

We presented looming grating stimuli over a range of spatial frequencies (0.017–0.272 cpd) to an age-matched cohort (n = 7; 14.4 ± 4.4sd weeks) of rd12 mice. We found significant responses at 0.068 cycles/degrees (Movie 7, Fig. 4g, left panel; p = 0.0001, n = 49 trials; signtest) and a similar response at lowest frequency tested that however did not reach significance (Supplementary Fig. 1a; p = 0.568, signtest, n = 49 trials, signtest; spatial frequency = 0.017 cycles/degree). These results are consistent with previously published measurements of the optokinetic reflex that reported a limit of spatial acuity below 0.2 cycles/degree^42^. Additionally we also found significant responses at 0.272 cycles/degree (Movie 8, Fig. 4g, right panel; p = 0.009, n = 49 trials, signtest). Responses to all stimuli consisted of a reduction in motor activity similar to the responses observed in control animals. Changepoint results were consistent across stimuli with a larger fraction of trials exhibiting an increase in changepoint rate (51%, 57%, 53% respectively at 0.017, 0.068, 0.272 cycles/degree) compared with trials exhibiting a decrease (34%, 34%, 36%). However reproducibility was lower than in visually intact animals (p = 0.008, permutation test comparing distributions of Δ#chp/s for visually intact and rd12 animals across all spatial frequencies) and the average increments in changepoints were only significant at 0.068 cycles/degree (Fig. 4h). Responses were also less reproducible across individuals (compared Supplementary Fig. 6b with Supplementary Fig. 5e).

### Behavioural habituation in mice with intact and degenerate retinas

As animals are presented with multiple repeats of the same or similar stimuli with no associated reward or punishment the salience of those stimuli is expected to “wash off”, reducing the drive for behavioural responses^43^. Indeed habituation to looming stimulation has been previously reported^31^. As habituation could potentially limit the number of effective trials that can be presented to an individual animal we asked to what extent this phenomenon affected our tests. To quantify habituation we focussed on those stimuli and animal groups associated with clearly detectable behavioural responses as with little (e.g. flash stimuli in “old” rd1, Fig. 4) or no responses this effect is not measurable (e.g. looming stimuli in rd1, Fig. 4e,f). Note that in order to pre-emptively mitigate the possibility of habituation by reducing expectation the presentation order of the stimuli throughout our study was block randomised (See Methods).

**Figure 5:**
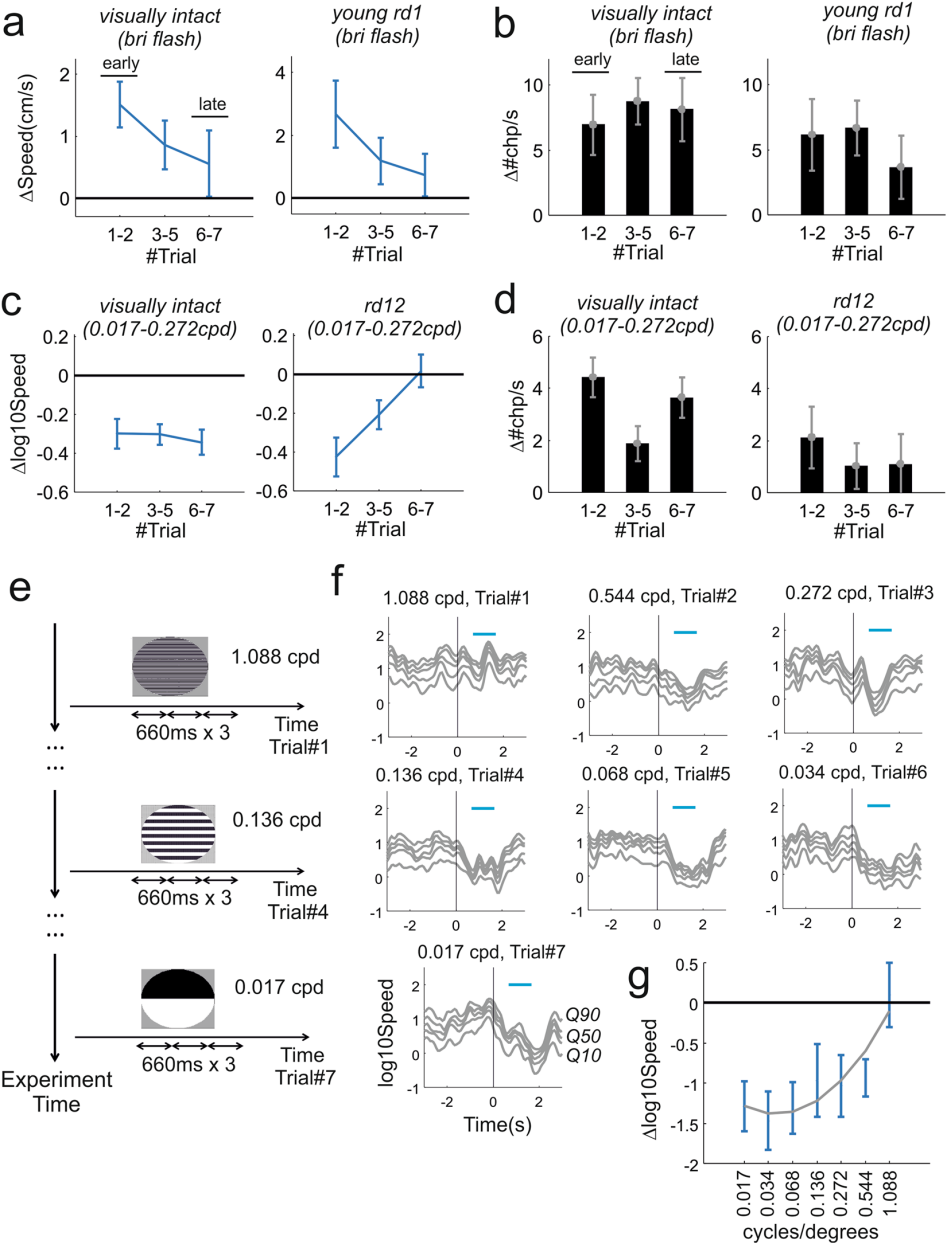
Behavioural habituation to repeated stimulation in visually intact and retinally degenerate mice. **(a)** Average changes in speed after stimulus onset as function of trial order for the highest intensity flash (data shown as mean ± sem). Represented in left and right panels are visually intact and “young” rd1 animals. Both groups exhibit a negative trend (visually intact: p = 0.049, n = 36 trials; young rd1: p = 0.039; n = 24 trials; comparisons between early and late trials, permutation test). **(b)** Difference between changepoint rate after and before stimulus onset (Δ#chp/s) as function of trial order during highest intensity flash for visually intact and young rd1 animals (respectively left and right panel). Neither group showed a significant trend (visually intact: p = 0.718, n = 36 trials; young rd1: p = 0.283; n = 24 trials; comparisons between early and late trials, ranksum test). (**c**,**d)** Like panels a and b but for looming responses in visually intact and rd12 animals. No significant trend was observed for visually intact animals (panel c: p = 0.482, n = 39 trials; panel d: p = 0.556, n = 39 trials). The rd12 groups exhibited gradual reduction in speed (p = 0.001, n = 21 trials) while no clear trend could be observed in changepoints (p = 0.401, n = 21 trials). (**e)** Alternative protocol based on systematic reduction of spatial frequency from highest to lowest (1.088, 0.544, 0.272, 0.136, 0.068, 0.034, 0.017 cycles/degree). Each spatial frequency was presented only on one trial and repeated three times (each repetition lasting 660 ms as previous tests reported in Fig. 1d; double arrows represent this time interval) during that trial as in^19^. (**f)** Average speed response for all spatial frequencies tested (n = 5 animals, 1 trial/animal). (**g)** Polynomial fit for changes in speed as function of spatial frequency (polynomial degree = 2; R^2^ = 0.263; same dataset shown in from panel f, data shown as mean ± sem). Consistent with results obtained by using the block randomised protocol (Fig. 3a–c) reduction in speed can be observed up to 0.544 cycles/degrees but not at 1.088 cycles/degrees.

During flash experiments changes in speed in animals with intact vision and in young rd1 were both trending towards lower amplitude in “late” trials (6^th^ and 7^th^ repetitions of the stimulus) compared with those recorded in “early” trials (1^st^ and 2^nd^ repetitions; Fig. 5a**;** p = 0.049, 0.039, respectively visually intact and young rd1, permutation test). The level of habituation to looming gratings was different in visually intact and rd12 animals. The former did not exhibit significant habituation (Fig. 5c, left panels; p = 0.482, permutation test) while the latter expressed a substantial reduction in speed changes across trials (Fig. 5c, right panel; p = 0.001). None of these effects were observed in changepoint rates indicating that while the amplitude of behavioural responses is reduced in “late” trials those responses are still present (Fig. 5b,d**;** visually intact: p = 0.718, 0.556 respectively flash and looming; rd1: p = 0.283; rd12: p = 0.401; permutation tests). Overall our data indicate that habituation can have measurable short-term effects (on the timescale of minutes) over the course of an experimental session.

In situations where many stimuli have to be presented, an alternative solution to counteract habituation could be to present stimuli in order by starting with those least likely to be detected. To test this possibility we repeated the estimation of spatial acuity in a new cohort of visually intact animals. We selected a wider set of spatial frequencies and presented them in decreasing order (Fig. 5e). In this way the minimally detectable stimulus (in this case threshold spatial frequency) will be associated with the strongest, non-habituated response, providing the clearest upper bound to estimate visual function. In agreement with the results in Fig. 3a,b this alternative protocol returned a limiting spatial acuity between 0.5 and 1 cycles/degrees (Fig. 5f,g).

## Discussion

Our results provide a proof of principle that systematic assessment of visually guided innate responses, such as freeze or escape, represents a promising tool to assess visual capabilities both in mice with intact visual system and in mice affected by different levels of visual impairment.

In the last few years, interest in visually driven innate behaviours has come of age (see e.g.^29,30,44^). The growth in interest in such behaviours has been attributable primarily to their application in studies of sensorimotor transformations and action selection. As these behaviours rely upon detection of a visual stimulus, and are readily elicited in untrained animals, they also have clear potential as a basis for characterising visual capacity. However several potential barriers stood in the way of realising that potential at the start of this project: 1) the degree to which behaviourally salient stimuli used to evoke innate responses could be parameterised was unknown; in other words, to what extent a systematic variation of a stimulus parameter (such as flash intensity or grating spatial frequency) can induce measurable differences in behavioural responses? 2) reliable, automated and objective methods for measuring innate behavioural responses were not well defined; 3) the intrinsic variability of innate behaviours may make them too unreliable as an indicator of whether a mouse had detected a visual stimulus; 4) innate responses could quickly habituate; 5) the extent to which visually driven behaviours appear in animals with visual impairment was unexplored. Below, by using our methodological and behavioural results as well as other recent studies, we discuss these issues:

### Parametrising behaviourally salient visual stimuli

Most studies on visually driven innate responses have focussed on looming stimuli in the form of a dark, enlarging, spot. While variants of this stimulus have been considered (see e.g.^19^) the general conclusion that defensive responses are strongly selective might have prevented a full exploration of the space of effective stimuli. Very recently it was shown that escape response to dark looming stimulus exhibits a smooth dependence on the contrast of a looming spot, indicating that negative contrast can be effectively parametrised^29^. Here we show that darkening looming stimuli do not represent the only stimulus class able to trigger defensive responses: isoluminant stimuli – black and white gratings whose average luminance matches a grey background - can reliably evoke freezing (Figs 1d, 3). Importantly we have shown that altering the spatial frequency of these gratings induces quantifiable changes in behavioural responses. Therefore those responses can be used to measure mouse spatial acuity. Moreover it is now reasonable to assume that changes in other grating characteristics e.g. contrast, colour etc. could be applied to quantify other visual features.

Although mice with advanced degeneration did not respond to gratings (rd1, Fig. 4a–f), we found that they did respond to another parameterisable stimulus: full field flashes. Responses to full field flashes have been previously shown to evoke behavioural responses whose magnitude depends on flash intensity^21^. Unexpectedly the responses we observed, consisting in sharp transient increases in activity, were different and opposite from the behavioural arrests observed by Liang and co-workers^21^. We note however that the environment in which those behavioural arrests were recorded, a narrow corridor, was substantially different from the open field arena used for this study. Therefore, a likely explanation for different behavioural outcomes is that different environments trigger diverse behavioural responses. Indeed a similar environmental effect, whereby the presence (or absence) of a shelter triggers escape (or freeze), is well known (compare e.g. results from^19^ – escape to^30^ – freeze).

### Methods for measuring innate responses

In this work we introduce a new method to track multiple body points and establish changepoint analyses to detect innate responses in behavioural time series. These methods allow detection of large changes in behaviours such as sudden locomotion arrests or initiations (see e.g. Movies 2, 7) as well as more subtle behavioural responses (see e.g. Movies 1, 4). Changepoint analysis is based on PELT algorithm and provides the optimal segmentation under a simple cost function^26^. It has only one free parameter, which can be quickly estimated from the data with the CROPS algorithm^45^ as shown in Methods, therefore results can be easily reproduced. Changepoint analysis is particularly useful for measuring how reliably behavioural responses occur across trials by simply subtracting the rate of changepoint before and after stimulus onset.

The results are robust in respect to the tracking algorithm used and the main results can be also replicated by using the more standard centroid method to track body position (Supplementary Figs 3 and 4). Therefore, while tracking multiple body parts has the advantage of providing a more graded representation of partial movements (Fig. 1), a more complete dataset to apply changepoint detection and is robust to occluded views of the animal body (e.g. when a shelter is present, see Movie 12), any tracking algorithm can potentially be used to detect the range of innate behaviours we describe in this study.

### Intrinsic variability of innate behaviours

Spontaneous behaviours can vary substantially between individuals. Thus, different animals exposed to the same threatening visual stimulus can employ diverse, and sometimes opposing (e.g. freezing or escape), behavioural strategies. However recent results indicate that different classes of visual stimuli can bias towards different types of innate responses and this bias is shared across individuals. Thus De Franceschi^20^ has shown that looming and sweeping dark spots can consistently drive mice to select either escape (for a looming spot) or freezing (for a sweeping spot). In this paper we describe another clear behavioural dichotomy as the same group of animals express sharp increments in speed after a flash presentation and freezing-like behaviours under loom + gratings stimuli (respectively Figs 2a, 3a).

Innate responses such as freezing and escape have been now described from several laboratories. While details of the motor sequences might vary (for example from Type I to Type II behaviours described in^31^) the occurrence of those behaviours is well established. In this work we reinforce this notion by showing that both looming evoked freezing-like behaviours and flash evoked increments in motor activity are maintained across different mouse strains. Moreover changepoint analysis can automatically detect both negative and positive changes in speed and therefore also addresses intrinsic variability of innate responses.

For each stimulus in each animal group we also reported the percentage of trials with positive, negative or no response (calculated as difference in number of changepoint before and after presentation of a visual stimulus). These numbers provide a measure of response reliability and can be used in the future to design adequately powered experiments (e.g. by using Experimental Design Assistant^46^ or other software) for testing treatments for retinal degeneration.

### Innate responses could quickly habituate

One obvious potential solution to inherent behavioural variability is to record responses to multiple stimulus presentations. However, when animals are presented with repeated sets of threatening stimuli, that turn out to be inconsequential, behavioural responses will quickly habituate^43^. Therefore habituation could represent a barrier to presenting large numbers of similar stimuli to the same animal as a method of addressing response variability and in order to describe responses to quantitative alterations in the stimulus (e.g. when estimating threshold spatial acuity). While we do observe some level of habituation in the amplitude of behavioural responses (Fig. 5a,c) those responses were still present across multiple repetitions (up to 7) and detectable by changepoint analysis (Fig. 5b,d). Thus when multiple stimuli must be presented to estimate detection thresholds, we find that habituation can be addressed either by randomising the presentation order of a limited set of stimuli (Fig. 3) or by presenting a wider set that starts from the least detectable stimulus (Fig. 5e–g).

In this study we did not investigate the potential for long-term habituation to the same set of stimuli presented on different experimental sessions. Others have described this phenomenon and reported it to be stimulus specific^43^. Our data are consistent with this view as we were able to record responses to two different sets of stimuli (flash and looming gratings) from the same visually intact animals on different days (Figs 2, 3). Thus, while it may not be sensible to re-test the same stimuli on a different occasion, simple modifications of the stimuli (e.g. changing orientation of looming gratings) that have been shown to enhance perception of novelty^47^ could allow re-testing.

### To what extent visually driven behaviours are conserved in animals with different levels of visual impairment?

A fundamental concern about the use of visually driven innate responses in describing sight loss is the extent to which they are conserved in animals with impaired visual function. Clearly an all-or-none situation whereby innate behaviours are either reliably expressed or fully abolished would only allow for a binary discrimination between animals with intact or impaired function. Given the wide spectrum of visual impairments occurring in different mouse models of ocular diseases and individual variability in degeneration and treatment efficacy this kind of binary discrimination would be of little use. In this study we used well characterised mouse models of retinal degeneration (see e.g.^36,48^ for rd1 and^42,49^ for rd12) and we showed that visually driven innate responses allow for a finer discrimination across different levels of visual impairments. Thus, quantitative differences (in flash responses between rd1 age groups, Fig. 4a) as well as qualitative differences (presence or absence of freeze responses to spatial stimuli, Fig. 4e–h) allowed for discrimination between severity and type of retinal degeneration. Our test is mainly intended to probe retinal function. However since degeneration of retinal output can potentially induce plastic changes in all retinal targets (see^50^ for a systematic assessment of retinal projections) it is also possible that those downstream changes also play a role in the suppression of visually evoked behaviours.”

## Conclusions

Our results indicate that systematic assessment of innate responses elicited by parameterisable stimuli represent a viable approach for measuring visual capabilities both in mice with intact visual function and in mouse models of retinal degeneration. Furthermore innate behaviours have several advantages over other established tests of visual function. The tests we propose are quick; can be performed on naïve mice; do not require specialist training for the experimenter; and the analyses are all automated. Compared with tests based on brainstem driven reflexes, such as optokinetic nystagmus or optomotor following, they capture higher limits in spatial acuity that match those obtained with the visual water task. Unlike the visual water task they do not require preliminary training sessions that can last several weeks in animals with limited vision^7^, can cause significant distress^14^ and introduce further variability in the results due to the learning process itself^16,17^. Finally, the use of innate behaviours allows for recording a wider repertoire of behavioural responses, such as speed increments after full field flashes and freeze after looming, increasing the experimenter’s ability to discriminate different levels of visual function.

## Methods

### Ethical statement

Experiments were conducted in accordance with the Animals, Scientific Procedures Act of 1986 (United Kingdom) and approved by the University of Manchester ethical review committee.

### Experimental set-up

We used an open field arena and recorded the animals with 2 programmable global shutter cameras (Chamaleon 3 from Point Grey) stably mounted on optical rails (Thorlabs; Fig. 1a). In order to avoid saturation due to changing light levels in the visual stimuli the camera lenses were covered with infrared cut-on filters (Edmund Optics) and fed with constant infrared light. Visual stimuli were delivered by a projector onto a rear projection screen. The flashes for the full field contrast stimuli were provided by two LEDs mounted inside the arena (LED Engin LZ4-00B208; controlled by T-Cube drivers, Thorlabs). The experiments were controlled by using Psychopy (version 1.82.01)^51^. Frame acquisition was synchronized with the projected images and across multiple cameras by a common electrical trigger delivered by an Arduino Uno board (arduino.cc) controlled by Psychopy through a serial interface (pyserial). Triggered acquisition through external TTL from the Arduino board was enabled on Chamaleon 3 cameras through FlyCapture2 software (from Point Grey). All movies were encoded as M-JPEG from RGB 1280 (W) × 1040 (H) images. For tracking RGB images were converted to grayscale. All stimuli were calibrated by using a spectroradiometer (Bentham Instruments) and retinal irradiance values for each photoreceptor were calculated by using Govardovskii templates^52^ and lens correction functions^53^ as previously described^54,55^. Summary information for all stimuli are provided in Tables 1, 2.

For all experiments we delivered 7 blocks of 2–3 different stimuli whose order was independently randomized within each block. We set inter-stimulus interval at 73 seconds. For each trial the video recording started 6 seconds before and ended 7 seconds after the stimulus onset. Framerate was set at 15 Hz and allowed us to collect 200 frames per trials.

### Animal resources, housing and handling

The following animals were used in this study: visually intact C57Bl/6 mice (n = 15, all male), retinally degenerate *rd1* (n = 20 mice, 19 male, 1 female) and *rd12* (n = 7, all male) mice. The *rd12* strain was provided by Prof. Robin Ali at University College London and is on a C57Bl/6 J background. This *rd12* strain originates from Jax Laboratories (JAX stock #005379). The visually intact C57Bl/6 mice were obtained from the Biological Services facility at University of Manchester, originally from Envigo. The *rd1* mice are on a mixed C57Bl/6 × C3H/HeNCrL background bred at the University of Manchester and were originally obtained from Prof. Mark Hankins at the University of Oxford.

All mice were stored in cages of 2–4 individuals and were provided with food and water ad libitum. During transfer between the cage and the behavioural arena we used the tube handling procedure instead of tail picking, as prescribed in^15^, in order to minimise stress and reduce variability across animals.Mice were kept on a 12:12 light dark cycle, with behavioural testing conducted during the light phase.

### Immunohistochemistry and cell imaging

Mice were culled by cervical dislocation, enucleated and eyes pierced with 24 gauge needle before being transferred to 4% paraformaldehyde in phosphate buffered saline (PBS) and stored overnight at 4 °C. Retinas were then dissected and permeabilised in PBS with 1% Triton-X for 3 × 10 mins. A background block incubation in PBS with 1% TritonX and 10% Donkey serum was conducted before retinas were incubated in primary antibody solution: PBS with 1% Triton-X with 2.5% donkey serum with 1:250 dilution of rabbit anti-short-wavelength sensitive (SWS) cone opsin antibody (AB5407, Merck Millipore) overnight at 4 °C. This anti-SWS cone opsin antibody is well-characterised for the fluorescent labelling of S-cones in the mouse retina^56–58^. Retinas were then washed in PBS with 0.2% TritonX for 4 × 1 hour, then incubated in secondary antibody solution: PBS with 1% TritonX and 2.5% Donkey serum with 1:250 dilution of donkey anti-rabbit Alexa 546 (A10040, Thermo Fisher) overnight at 4 °C. Retinas were washed in PBS with 0.2% TritonX for 4 × 1 hour, then washed in distilled water for 10 mins before being mounted ganglion cell-side up on microscope slides using Prolong Gold anti-fade mounting reagent (Thermo Fisher) and left to cure overnight at room temperature. Slides were stored at 4 °C until imaging.

Images were acquired using a Leica DM2500 microscope and Leica DFC365 FX camera with CoolLED pE300-white light source. Images were collected using Leica Aplication Suite Advanced Fluorescence6000 (LAS AF6000) software. The filters used were Chroma ET Y3 filter set (excitation = 545 nm, emission = 610 nm). For x20 air objective; exposure time = 100 ms and gain = 2. For x100 oil objective; exposure time = 50 ms and gain = 2. Global enhancements of brightness and contrast were applied equally to all 20× (+40% brightness and +20% contrast) and 100× (+20% brightness and +40% contrast) images using ImageJ in Supplementary Fig. 7.

### Mouse tracking

We first isolated the mouse body by using a static background acquired at the beginning of each experiment before introducing the animal into the arena. The initial subtraction was refined by an opening operation (erosion followed by dilation) that removed mouse excrements that occasionally accumulated on arena floor during the experiment. These operations allowed us to extract a tightly bounded box around the animal body that sped up subsequent calculations.

We found that the classic Harris corner detector was effective in detecting most relevant landmarks such as ears, snout tip, paws extremities, tail base and whiskers. However when just applied to the original image it only returned a partial list of features per image. In order to increase the yield of Harris detector we applied it to the associated 6 levels image pyramid representation (sample factor = 1.25 with 13 points Gaussian smoothing, σ = 1.25). This returned a dense labelling of body landmarks as shown in Fig. 1b (bottom panels).

In order to quantify landmark speed the landmarks between two successive images were initially matched by using Pearson’s correlation. Typically not all matches were correctly assigned and incorrect assignments could introduce artefactual jumps in the behavioural time series. To address this problem we applied the following algorithm to identify and reject outliers. We estimated a partial Procrustes superimposition (rotation + translation) between matched landmarks coordinates. The estimation was performed by employing the RANSAC algorithm^59^ that allowed us to reject outliers given a fixed error threshold (inlier threshold = 10 pixels). While a single linear transformation was effective in capturing movements with consistent direction and amplitude across the whole mouse body (Fig. 1b, right panel) it failed to capture movements where different body parts where displaced in different directions and/or with different amplitudes. In order to capture the whole gamut of mouse movements we extended our approach by combining clustering and Procrustes analysis. Thus landmark coordinates were clustered via the *kmeans* ++ algorithm^60^ by systematically varying K, the number of clusters (K = [1, 5]; for each value of K clustering was performed 10 times to avoid suboptimal solutions). Procrustes partial superimposition was then separately estimated for each cluster (Fig. 1b, centre panel). To identify the optimal number of clusters we then compared the residuals by using as criterion the Minimum Description Length^61^ that allowed us to incorporate model complexity (i.e. the number of clusters) and the cost of different number of outliers. The description length of each model was defined as:

1 $$DL={L}_{which\_model}+{L}_{outlier}+{L}_{model}+{L}_{residuals}$$

and

2 $$\begin{array}{ccc}{L}_{which{\rm{\_}}model} & = & Nlo{g}_{2}(K+1)/ln(2)\\ {L}_{outlier} & = & {N}_{outlier}lo{g}_{2}({N}_{x}{N}_{y})/ln(2)\\ {L}_{model} & = & \frac{3}{2\,{\rm{l}}{\rm{n}}(2)}lo{g}_{2}(\prod _{i=1}^{K}{N}_{i})\\ {L}_{residuals} & = & \sum _{i=1}^{K}\{\begin{array}{c}\frac{{N}_{i}}{2}[{\rm{l}}{\rm{n}}(2\pi {\sigma }_{{x}_{i}}^{2})+\,{\rm{l}}{\rm{n}}(2\pi {\sigma }_{{y}_{i}}^{2})]+\sum _{j=1}^{{N}_{i}}\frac{{({x}_{i,j}-{\mu }_{{x}_{i}})}^{2}}{2{\sigma }_{{x}_{i}}^{2}}+\sum _{j=1}^{{N}_{i}}\frac{{({y}_{i,j}-{\mu }_{{y}_{i}})}^{2}}{2{\sigma }_{{y}_{i}}^{2}}\,\end{array}\}\end{array}$$

where *N*_*x*_*N*_*y*_ represents the area of the bounding box around the animal, *N*_*outlier*_ the total number of outliers, *N*_*i*_ the number of inliers for the *i*^*th*^ cluster, *x*_*i*,*j*_ the *j*^*th*^ residual of the *i*^*th*^ cluster on the x direction. The first term of Eq. 1, *L*_*which_model*_ accounts for the number of *nats* (natural bits) required to identify each point as outlier or belonging to the *i*^*th*^ cluster. The second term costs the outliers as bounded integers in the area bounding box. The third term penalizes for the number of clusters and the factor 3 corresponds to the number of parameters required to define the partial Procrustes superimposition for each cluster (1 for rotation, 2 for translation). The final term uses the fact that the cost of encoding residuals can be approximated by the negative log likelihood.

In order to compare results obtained with this technique with more standard tracking of mouse body centre we also performed additional tracking to evaluate whole body movements. To extract mouse body centre in each frame we first performed background subtraction followed by opening operation as described above. All pixels below a pre-specified threshold were inactivated. The body centre was then calculated as median coordinates of the remaining pixel positions.

### Generation of movement time series

We used the distribution of landmarks speed to generate multidimensional behavioural time series. We first calculated the speed of each landmark between two consecutive frames (Fig. 1b, bottom panels). From the distribution of landmarks speed we then calculated the 10^th^, 30^th^, 50^th^, 70^th^, 90^th^ speed quantiles for each frame (Fig. 1b, top panels; respectively Q10 to Q90). Each of these quantiles, estimated separately for each of the two cameras used (Fig. 1a), defined a dimension of the multivariate time series (Fig. 1c). For all subsequent analyses the quantiles were then averaged across the cameras (Figs 2a–4a,e,g).

### Changepoints detection

In order to find the number and the location of the changepoints we used the Pruned Exact Linear Time method (PELT) (Killick *et al*.^26^). PELT is designed to minimize a penalised cost function of the following form:

3 $$\sum _{i=1}^{m+1}[C({y}_{({\tau }_{i-1}+1):{\tau }_{i}})]+\beta m$$

where *C* is the cost function, *τ* represents the changepoints, *m* is the number of changepoints and β is the penalty for each additional change. This method has two main advantages: it calculates the global optimum of (3) and it does so in a computational cost, under mild conditions, that scales linearly with the number of data points. We define the cost function *C* as follows:

4 $$C({y}_{({\tau }_{i-1}+1):{\tau }_{i}})=\sum _{{\tau }_{i-1}+1}^{{\tau }_{i}}{({y}_{i}-{y}_{({\tau }_{i-1}+1):{\tau }_{i}})}^{2}$$

where <y> represents the average speed between two successive changepoints. The β term in (3) represents a penalty against overfitting. Popular choices for the penalty function include the Schwarz Information Criterion (Schwarz, 1978). Several authors also proposed different solutions to this problem by adaptations of the SIC to the problem of multiple changepoint analyses (Zhang and Siegmund, 2007). As a general consensus lacks about which criterion is more convenient for a given dataset we devised a method to automatically derive a suitable penalty range from the data. Therefore, instead of specifying a penalty value, for each trial we systematically scanned across a wide range of β values by using the Changepoint for a Range Of PenaltieS (CROPS) algorithm (Haynes *et al*., 2017). The CROPS algorithm efficiently finds all the sets of changepoints whose segmentations are optimal under some choice of β within an interval [βmin, βmax] by sequentially dividing this interval until guaranteed convergence. We then used the output of this algorithm, the number of changepoints as function of penalty values, to fit a piecewise linear model represented by two lines intersecting at a “knee” point (Supplementary Fig. 1b). The knee point marks the transition between under and over fitting and thus represents an ideal choice for the penalty. The segmented relationship between the number of changepoints and the penalty value β was modelled following (Muggeo, 2003) as

5 $$E[m]=a\beta +b{(\beta -\psi )}_{+}+c$$

where parameters *a* and *b* represent respectively the slope of the left line segment and the difference in slopes between the left and the right line, ${(\beta -\psi )}_{+}$equals 1 for $\beta  > \psi$ and 0 otherwise, and *ψ* represents the knee point. By using the iterative estimation proposed by (Muggeo, 2003) we then obtained a maximum likelihood estimation of all the parameters including the knee point (Supplementary Fig. 1b). We repeated this procedure across all trials to obtain the distribution of knee values for our datasets that provided a suitable range for choosing the penalty value β (Supplementary Fig. 1c).

### Statistics

Statistics derived from tracking data were applied either to the raw multivariate time series (for full field flash) or to a truncated log transformation of these data (for loom + gratings). The former was positively skewed, the second negatively skewed. Accordingly changepoints applied to the raw data was more biased towards detecting positive changes in movement, such as flights, the second towards negative changes such as freezing.

The significance of behavioural responses to individual stimuli was assessed by using the two-tailed signtest (in Figs 2a–4a,e,g). The test statistic was obtained by subtracting the mean landmarks speed after stimulus onset from that recorded before the stimulus onset. Speed values were estimated in time windows of equal duration (0.53 seconds for both looming and flash). Comparison between flash responses was performed on the same speed distributions by using the two tailed Wilcoxon rank-sum test (e.g. between “young” and “old” rd1 mice, Fig. 4a). Statistical significance for changepoints was calculated by subtracting the rate of change points before - *#chp/s(pre)* - and after - *#chp/s(post)* - stimulus onset using the same time windows and applying a two-tailed Wilcoxon rank-sum test. Thus the difference in changepoint rate (Δ#chp/s in Figs 2b–4d,f,h) was calculated as Δ#chp/s = *#chp/s(pre) - #chp/s(post)*. Statistical significance of intensity dependence for flash responses shown in Figs 2b, 4d was calculated from the same data by using Kruskal-Wallis test.

In order to test habituation both in speed and changepoint responses we used permutation tests. First the difference in speed responses (or changepoint responses) was calculated between early and late trials (Fig. 5a,b). Then this difference was recalculated 100000 times by shuffling the order of the stimulus trials and a p-value was determined as the fraction of instances in which these surrogate values were larger than the original statistic.

All statistical tests presented in the main and supplementary figures are detailed in Supplementary Tables 1, 2.

### Software resources

Changepoint analyses were performed in R^62^. The code for changepoint analysis, including the CROPS method, can be found in the ‘changepoint’ package^63^. For knee estimation we used the ‘segmented’ package^64^. The code for mouse tracking was written in MATLAB (Matworks, Natick, Massachusetts, USA).

## Supplementary information

Supplementary Information

movie 1

movie 2

movie 3

movie 4

movie 5

movie 6

movie 7

movie 8

movie 9

movie 10

movie 11

movie 12

## Data Availability

Data will be made available on request. Contact riccardo.storchi@manchester.ac.uk.
