# Supplementary material for: Measuring vision using innate behaviours in mice with intact and impaired retina function
Source: Sci Rep. 2019 Jul 17;9:10396. doi: 10.1038/s41598-019-46836-y (PMC6637134; doi:10.1038/s41598-019-46836-y)
Supplement: Supplementary file 1 — Supplementary Information [file 41598_2019_46836_MOESM1_ESM.docx]

**Supplementary Information:**

**Measuring vision using innate behaviours in mice with intact and impaired retina function**

R. Storchi*, J. Rodgers *, M. Gracey*, F.P. Martial*, J. Wynne*, S. Ryan**, C.J. Twining***, T.F. Cootes*, R. Killick** and R.J. Lucas*

*Faculty of Biology, Medicine and Health | University of Manchester, Manchester, UK

**Department of Mathematics and Statistics | Lancaster University, Lancaster, UK

***School of Computer Science | University of Manchester, Manchester, UK

**Contact: riccardo.storchi@manchester.ac.uk**

**Table of Contents:**

**Supplementary Figures**

- **Supplementary Figure 1**
- **Supplementary Figure 2**
- **Supplementary Figure 3**
- **Supplementary Figure 4**
- **Supplementary Figure 5**
- **Supplementary Figure 6**
- **Supplementary Figure 7**

**Movie Captions**

**Statistics Summary**

- **Supplementary Table 1**
- **Supplementary Table 2**

**Supplementary Figures:**

**
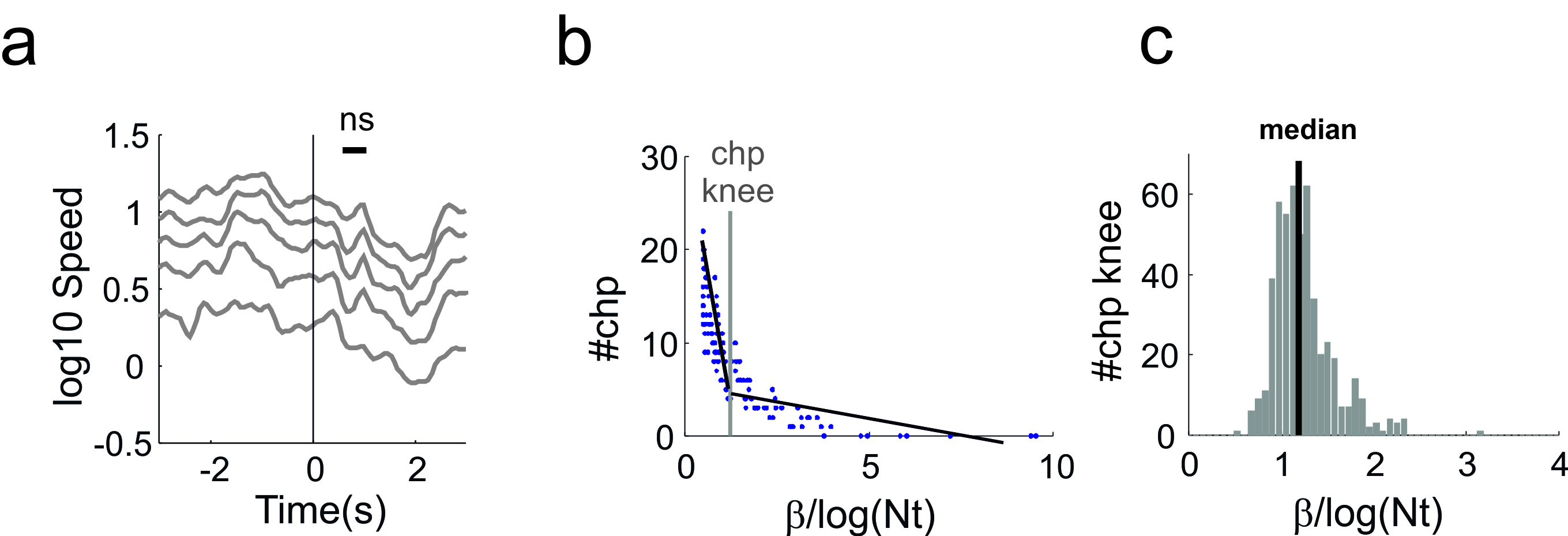
**

**Supplementary Figure 1: Looming response at 0.017 cpd for rd12 animals & data driven estimation of penalty value for changepoint detection. a)** Movement response, in log scale, to 0.017 cycles/degree gratings. **b)** Individual trial example where the number of changepoints (“#chp”, blue dots) across the dimensions of the multivariate time series are plotted as function of penalty. The knee (“chp knee”, grey vertical line) is estimated by fitting a segmented function (black lines meeting at the knee point, see Methods) between number of changepoints and penalty values. **c)** Distribution of knee values for our dataset. The median (black line) is then used for all further changepoint analyses.


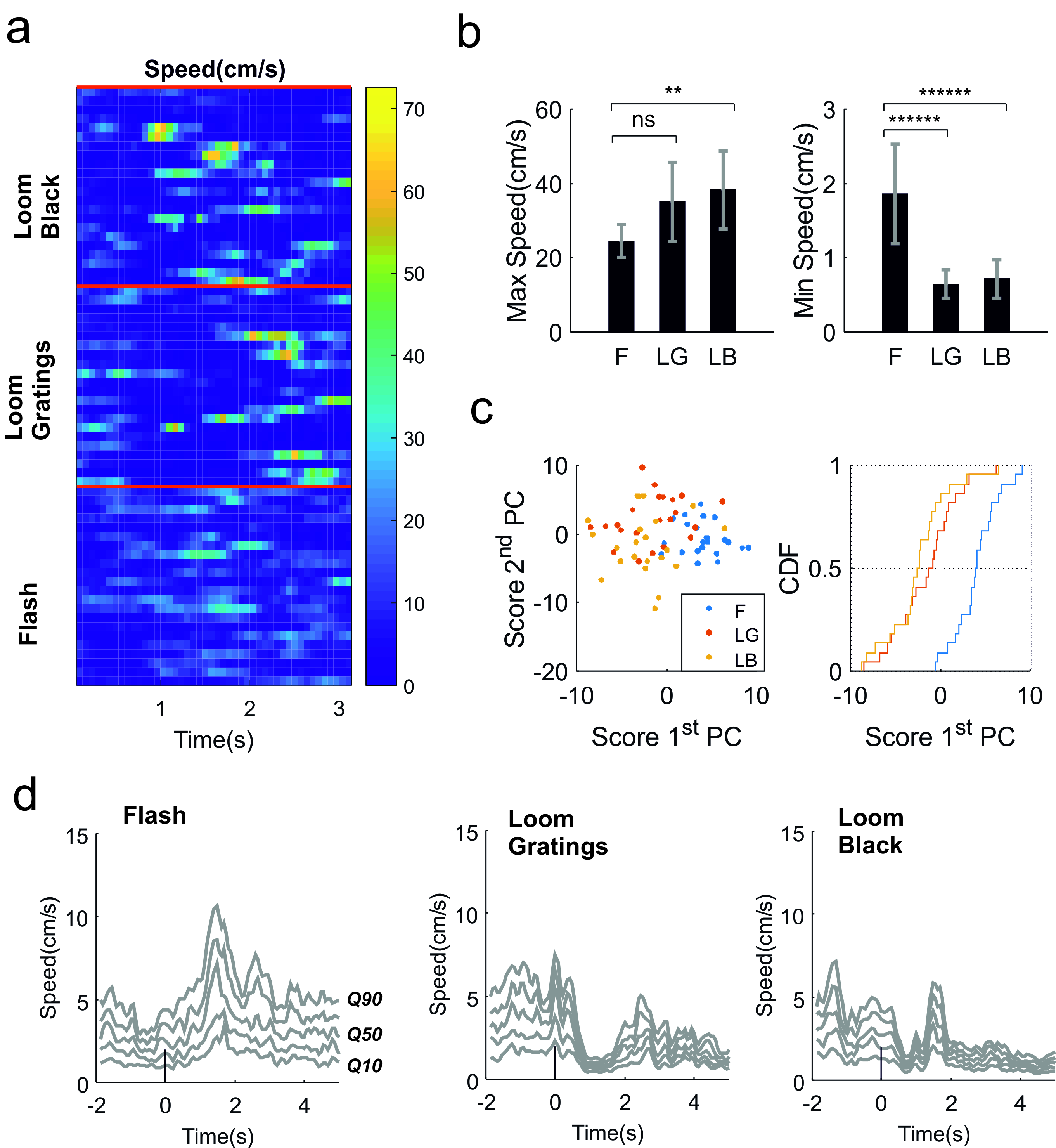


**Supplementary Figure 2: a)** Heatmap representing running speed for individual trials. Trials are grouped along the y axis according to the visual stimulus they relate to (from bottom to top: Flash, Loom+Gratings, Black Looming; 2 trials per animal were collected for a total of 22 trials per stimulus type). **b)** Maximum and minimum speed for each trials reported respectively in left and right panels. Maximum speed after flash is significantly lower than after black looming (n = 22 trials; p = 0.076, 0.0083 respectively flash vs loom+gratings and flash vs black looming, Wilcoxon rank-sum test). Minimum speed is significantly higher for flash compared with both looming stimuli (n = 22 trials; p = 6*10^-7^, 2*10^-6^ respectively flash vs loom+gratings and flash vs black looming, Wilcoxon rank-sum test). Each bar represents a different stimulus type (F = Flash, LG = Loom + Gratings, LB = Looming Black). **c)** Results from principal component analysis of single trials responses reported in panel **a**. On the left panel the scores for the first two principal components are reported. Dots represent individual trials (F = Flash, LG = Loom + Gratings, LB = Looming Black). On the right panel we report the empirical cumulative distribution function (CDF) for the different stimulus types (same colour convention of the left panel). d) Average speed quantiles for Flash, Loom+Gratings and LoomBlack stimuli (respectively left, middle and right panels). * p < 0.05, ** p < 0..01, *** p < 0.005, **** p < 0.001, ***** p < 0.0005, ****** p < 0.0001, ns = not significant.


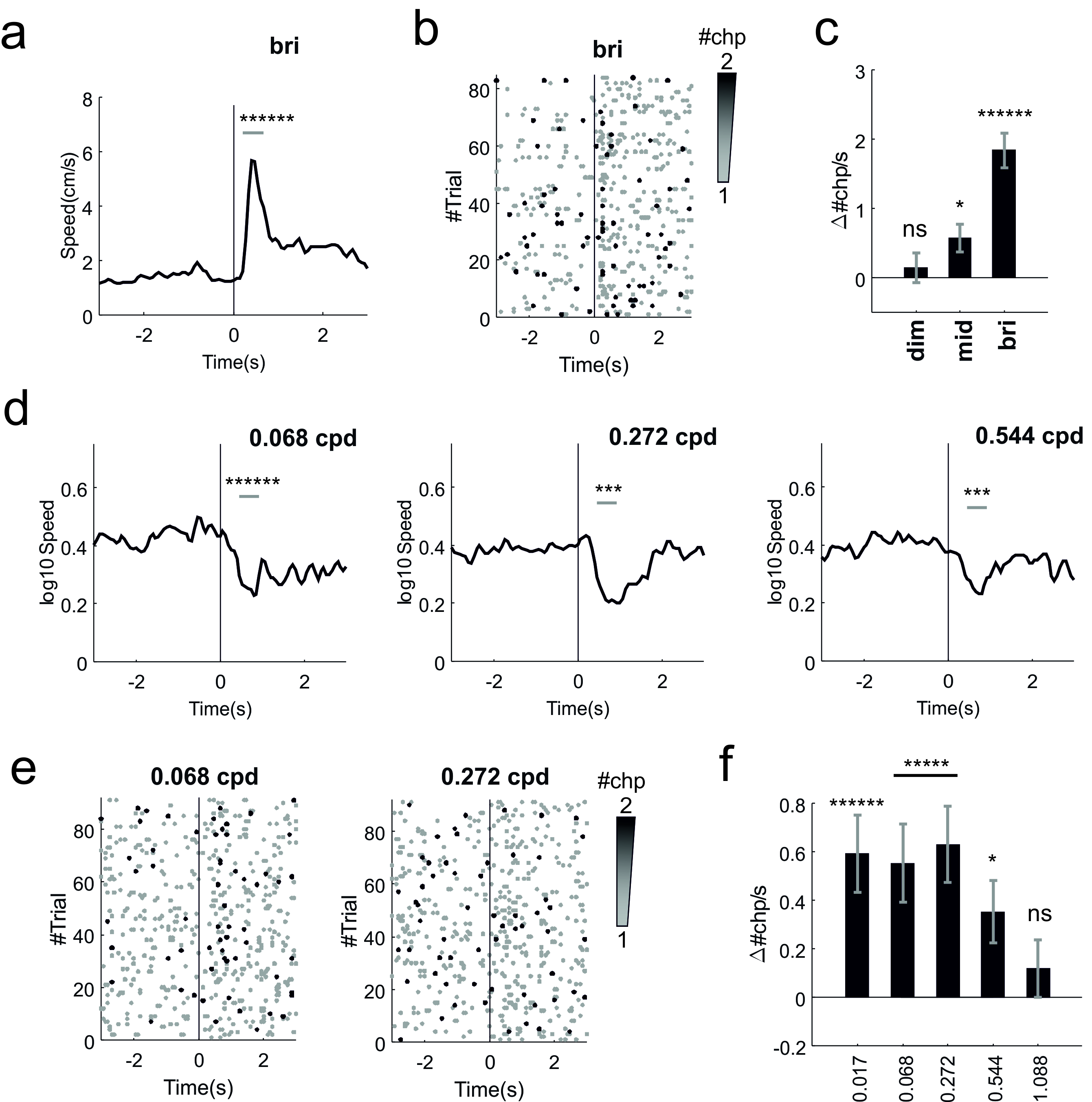


**Supplementary Figure 3:** All the results in this figure are obtained from the same datasets of Figure 2 and 3 but by using standard centroid tracking (See Methods). **a)** Average speed response to the bright flash. **b)** Changepoint detection across individual trials during presentation of the brightest flash. **c)** Number of changepoints detected after the bright flash subtracted by the number of changepoints detected before the stimulus onset (Mean ± SEM). **d)** Average log speed response to the Looming+Gratings stimulus at different spatial frequencies (frequencies reported at the top of each panel). **e)** Changepoint detection across individual trials (spatial frequencies of the gratings reported at top of each panel). **f)** Number of changepoints detected after the Looming+ Gratings stimulus subtracted by the number of changepoints detected before the stimulus onset (Mean ± SEM). Spatial frequencies reported along the x axis. * p < 0.05, ** p < 0..01, *** p < 0.005, **** p < 0.001, ***** p < 0.0005, ****** p < 0.0001, ns = not significant.


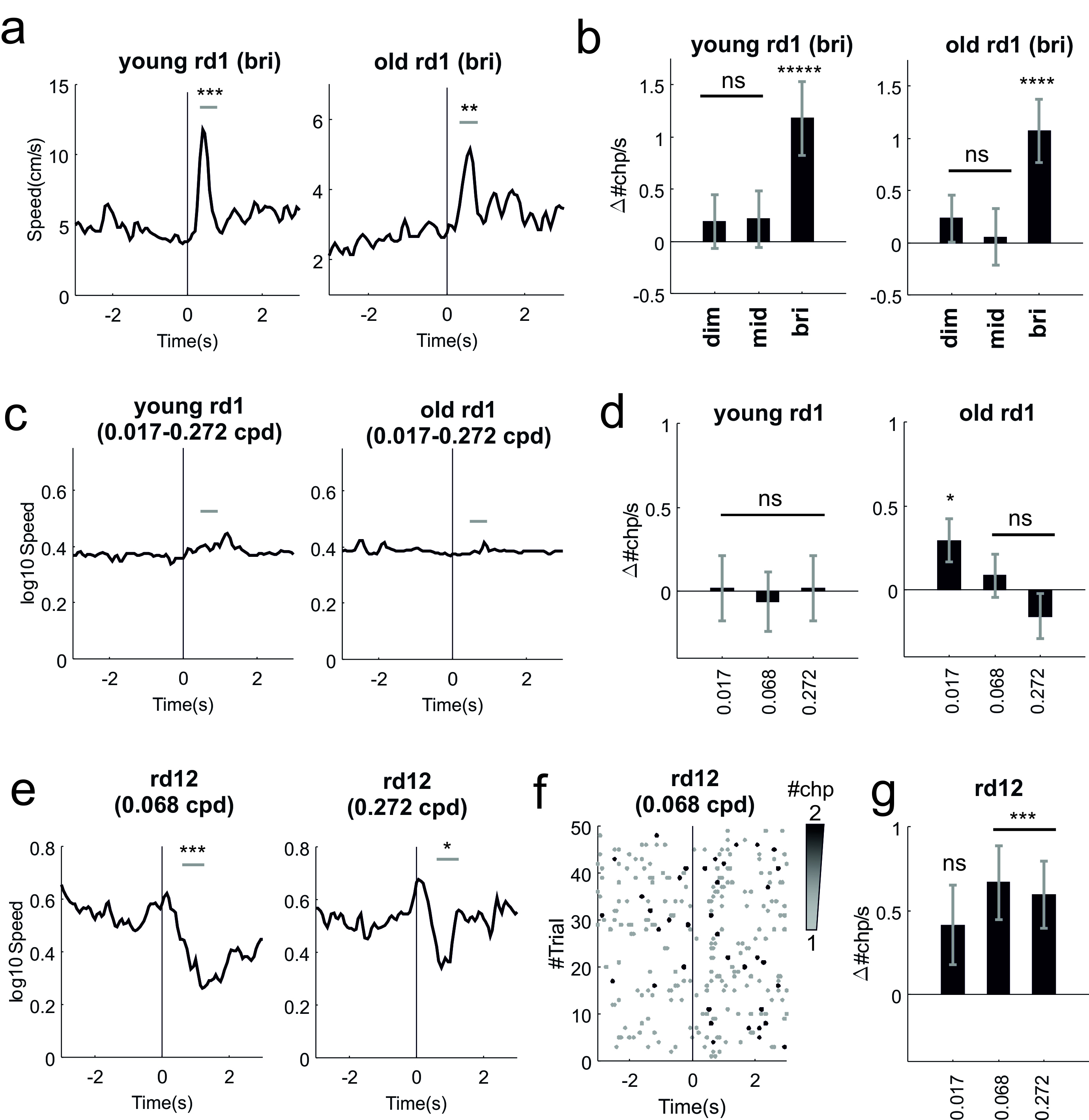


**Supplementary Figure 4:** All the results in this figure are obtained from the same movies of Figure 2 and 3 but by using standard centroid tracking (See Methods). **a)** Average speed response to the bright flash for the groups of “young” and “old” rd1 mice (respectively left and right panel). **b)** Number of changepoints detected after the bright flash subtracted by the number of changepoints detected before the stimulus onset (Mean ± SEM) for the groups of “young” and “old” rd1 mice. **c)** Average log speed response to the Looming+Gratings stimulus across a range of spatial frequencies (frequencies reported at the top of each panel). **d)** Number of changepoints detected after the Looming+ Gratings stimulus subtracted by the number of changepoints detected before the stimulus onset (Mean ± SEM). Left and right panels correspond respectively to the groups of “young” and “old” rd1 mice. Spatial frequencies reported along the x axis. **e)** Average log speed response to the Looming+Gratings at different spatial frequencies for the group of rd12 mice (spatial frequencies reported at the top of each panel). **f)** Changepoint detection across individual trials (spatial frequencies of the gratings = 0.068 cpd) for the group of rd12 mice. **g)** Number of changepoints detected after the Looming+ Gratings stimulus subtracted by the number of changepoints detected before the stimulus onset (Mean ± SEM) for the group of rd12 mice. Spatial frequencies reported along the x axis. * p < 0.05, ** p < 0..01, *** p < 0.005, **** p < 0.001, ***** p < 0.0005, ****** p < 0.0001, ns = not significant.


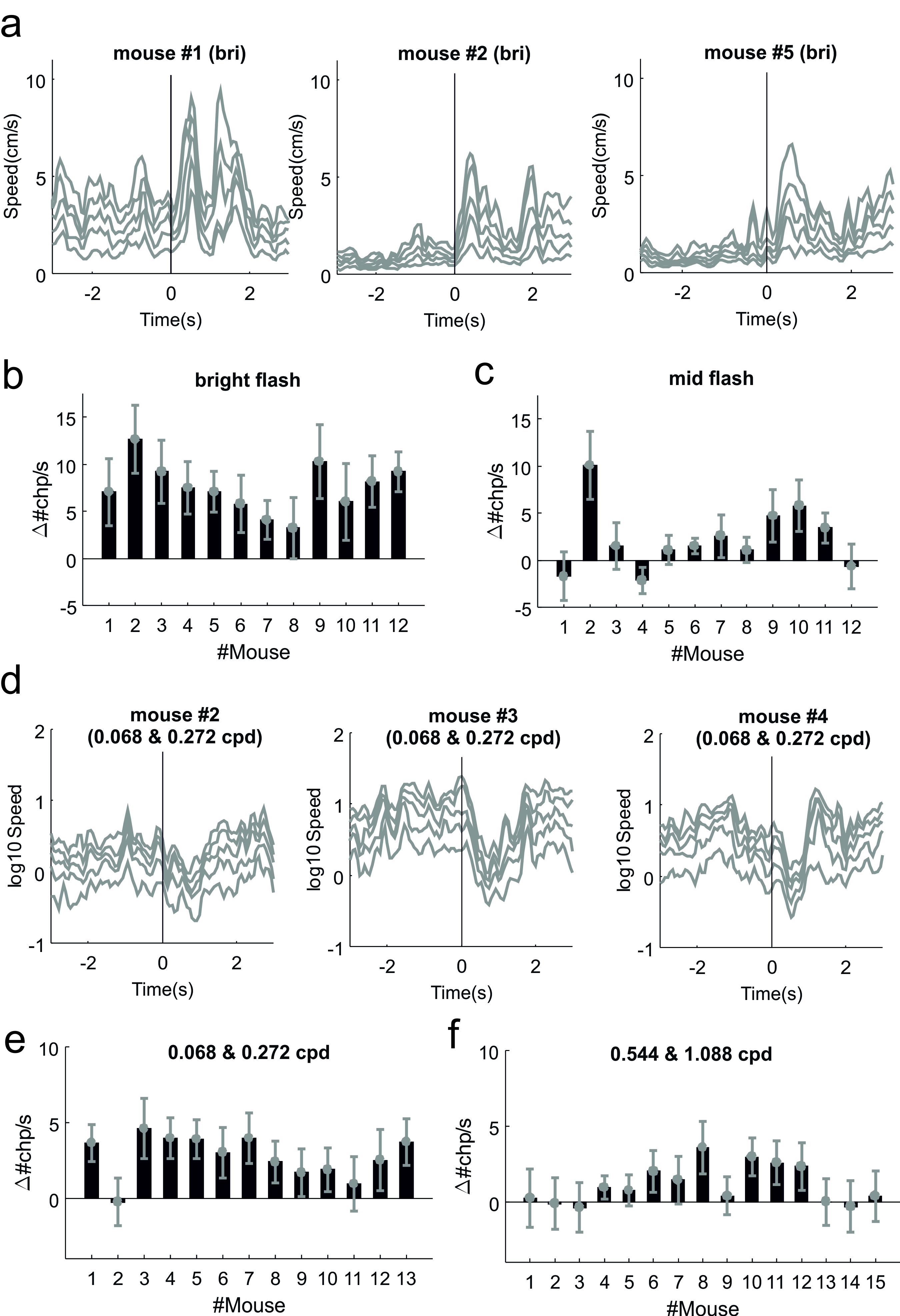


**Supplementary Figure 5:** All data in this figure are obtained from the group of visually intact animals. **a)** Average quantile speed to the bright flash for individual animals (mouse 1, 2, 5 respectively left, middle and right panel). **b)** Number of changepoints detected after the bright flash subtracted by the number of changepoints detected before the stimulus onset (Mean ± SEM). Results provided for individual animals. Animal ID reported on the x axis. **c)** Same as in panel **b** for the mid intensity flash. **d)** Average quantile log speed response to the Looming+Gratings from individual animals (mouse 2, 3, 4 respectively left, middle and right panel) for a range of spatial frequencies (0.068 & 0.272 cpd) at which animals respond most effectively. **e)** Number of changepoints detected after Looming+Gratings subtracted by the number of changepoints detected before the stimulus onset (Mean ± SEM) for the same a range of spatial frequencies reported in panel **d**. Data shown for individual animals; animal ID reported on the x axis. **f)** Same as panel **e** but for a range of spatial frequencies around the highest limit for spatial detection.


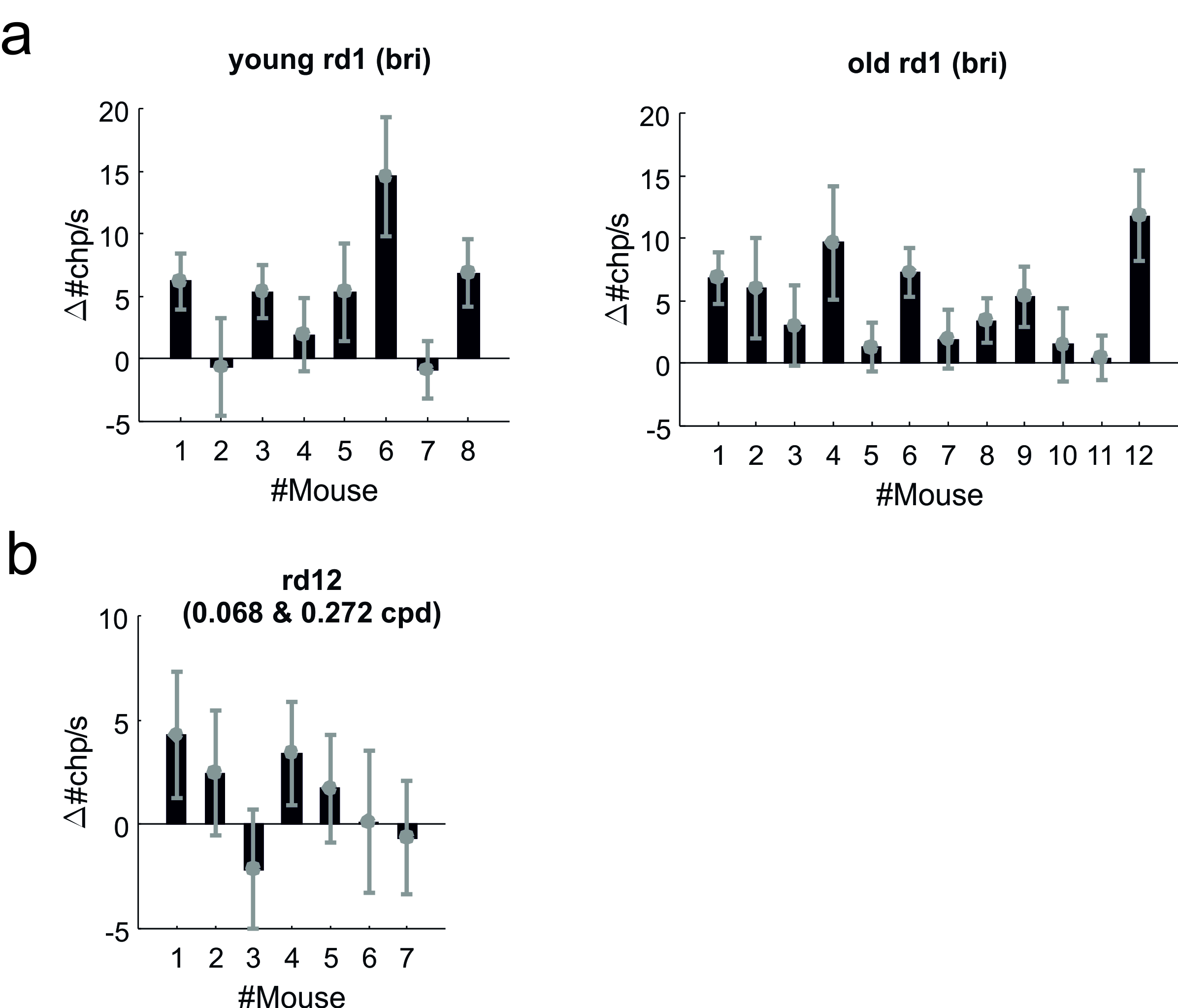


**Supplementary Figure 6: a)** Number of changepoints detected after the bright flash subtracted by the number of changepoints detected before the stimulus onset (Mean ± SEM). Data shown separately for the groups of “young” and “old” rd1 animals (respectively left and right panels). **b)** Same as **Supplementary Figure 5e&f** but for the group of rd12 animals.


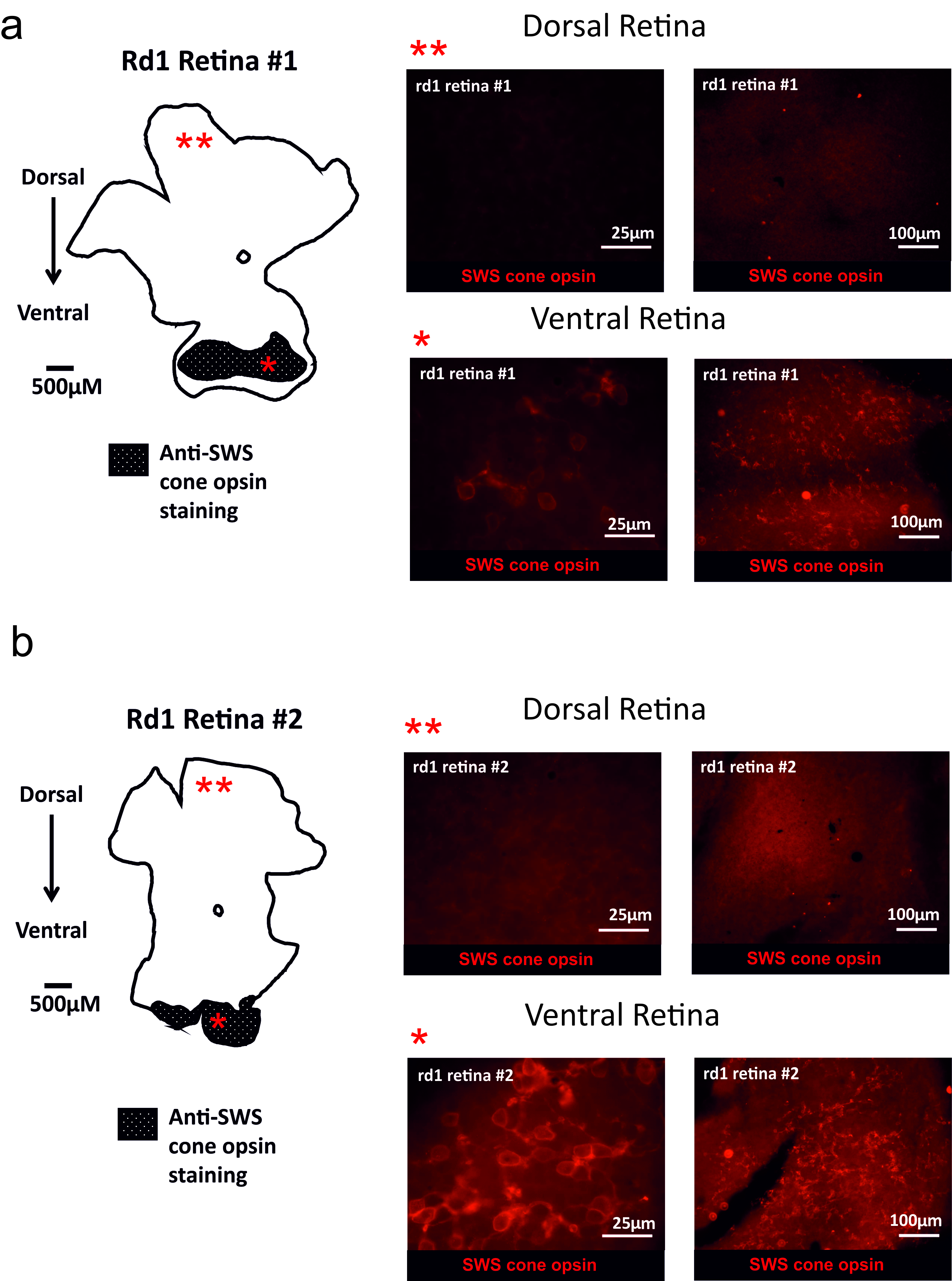


**Supplementary Figure 7:** Immunostaining of retinal wholemounts from “old” cohort of *rd1*mice demonstrated surviving S-cones in the ventral regions for two different retinas shown in **a)** and **b)**. For each retina, a diagram demonstrating the pattern of anti-SWS cone opsin staining across the wholemount is displayed on the left. Example micrographs from the dorsal (top) and ventral (bottom) of each retina collected at low (middle) and high (right) magnification are shown. Asterisk shows the location of micrographs in diagram of retinal wholemounts (** Dorsal, * Ventral). All micrographs show anti-SWS cone opsin staining. For all retinas we examined, we consistently observed a dense area of cells labelled with anti-SWS cone opsin antibody in the peripheral ventral region. Anti-SWS cone opsin staining of cells was not observed in the dorsal regions.

**Movie Captions**

**Movie 1:** Increase in movement by parts of the mouse body; here occurring as a change in rearing posture elicited by “bright” flash (stimulus occurring at time 0). Top left panel represents the quantile of the speed distribution (10^th^, 30^th^, 50^th^, 70^th^, 90^th^ quantiles - respectively Q10 to Q90) for the two cameras used for the study (for ease of representation here only the movie from camera1 is shown). Bottom left panels represents single changepoints associated with quantile time series (Q10 to Q90 for the two cameras). Pooled changepoints (see Methods) are also reported at the top line (“Pooled”).

**Movie 2:** Increase in full body movements elicited by “bright” flash; here the flash initiates locomotor activity. Panels displayed as in Movie 1.

**Movie 3:** Decrease in full body movements elicited by loom+gratings stimulus (gratings at 0.272 cycles/degree); here the gratings (occurring at time 0) arrests mouse locomotion in freeze-like behaviour. Panels displayed as in Movie 1.

**Movie 4:** Decrease in movements by parts of the mouse boy elicited by loom+gratings stimulus (gratings at 0.0.068 cycles/degree); here the animal, engaged in stationary exploration, produces a freeze-like response. Panels displayed as in Movie 1.

**Movie 5:** Increase in movement in a “young” rd1 mouse elicited by “bright” flash. Panels displayed as in Movie 1.

**Movie 6:** Increase in movement in an “old” rd1 mouse elicited by “bright” flash. Panels displayed as in Movie 1.

**Movie 7:** Decrease in movement in an rd12 mouse elicited by loom+gratings stimulus (gratings at 0.068 cycles/degree). Panels displayed as in Movie 1.

**Movie 8:** Decrease in movement in an rd12 mouse elicited by loom+gratings stimulus (gratings at 0.272 cycles/degree). Panels displayed as in Movie 1.

**Movie 9:** Increase in movement elicited by “bright” flash stimulus (stimulus onset at time 0) in visually intact animals. Note that in this experiment a shelter is provided. The flash does not evoke escape to the shelter. On the left panel we report quantile speed (Q10 to Q90) from one camera.

**Movie 10:** Mixed freeze-escape response elicited by loom+gratings stimulus (gratings at 0.068 cycles/degree). Panels displayed as in Movie 9.

**Movie 11:** Escape response elicited by the black looming stimulus. Panels displayed as in Movie 9.

**Movie 12:** The movie shows the tracking of an animal whose body parts are partially occluded by the shelter. In spite of occlusion the visible body parts are tracked throughout the recording.

**Statistics Summary:**

**Supplementary Table 1:** Summary of all statistical tests reported in Figures 1-5.

**Supplementary Table 2:** Summary of all statistical tests reported in Supplementary Figures 1-7.
